# Supplementary material for: Suicide methods among Brazilian women from 1980 to 2019: Influence of age, period, and cohort
Source: PLoS One. 2024 Dec 13;19(12):e0311360. doi: 10.1371/journal.pone.0311360 (PMC11642912; doi:10.1371/journal.pone.0311360)
Supplement: S1 Table — (DOCX) [file pone.0311360.s001.docx]

**S1 Table**. GATHER checklist of information that should be included in reports of global health estimates

**Objectives and funding**

Item1- Define the indicator(s), populations (including age, sex, and geographic entities), and time period(s) for which estimates were made.

R- Mortality rates for suicides among women by method (HSS, AUT, and FA) were calculated, stratified by Brazilian regions (North, Northeast, Southeast, South, and Center-West), by age group (10-14, 15-19 up to 80 or more years), by period (1980-1984, 1985-1989 up to 2015-2019), and by cohort (1900-1904, 1905-1909 up to 2005-2009).

Item2- List the funding sources for the work.

R- This study received financial support from the Nation al Council for Scientific and Technological Development (CNPq- 306652/2022-6). and the Coordination for the Im provement of Higher Education Personnel (CAPES, Funding Code 001).

***For all data inputs from multiple sources that are synthesised as part of the study:***

**Data inputs**

3-Describe how the data were identified and how the data were accessed.

R-The data used in this study were freely accessed from the Mortality Information System of the SUS Department of Informatics (SIM/DATASUS). This system is universally accessible. SIM/DATASUS is the information system of the Brazilian Ministry of Health ([DATASUS – Ministério da Saúde (saude.gov.br)](https://datasus.saude.gov.br/)), which provides death records for all Brazilian states and municipalities since 1979. In the present study, the microdata for each Brazilian region was collected annually from 1980 to 2019. The microdata is available in dbc format and was converted to dbf format using the Tabwin program version 4.15 for Windows provided by the Brazilian Ministry of Health. After converting the data to dbf format, the death records for each year (1980 to 2019) were aggregated for each of the Brazilian regions using the R software (version 4.1), extracting only death records of females aged 10 years and older.

Population data for mortality rate estimates were also obtained from DATASUS (http:// www2.datasus.gov.br/DATASUS/index.php?area=0206&id=6942) in the sociodemographic and economic data section, based on a demographic census from 1980, 1991, 2000 and 2010. The Brazilian Institute of Geography and Statistics estimated populations projections on July 10 of the intercensal years [20].

4- Specify the inclusion and exclusion criteria. Identify all ad-hoc exclusions.

R- This study only included death records of women aged 10 years and older with the coding listed in the table below.

**Table 1.** Death records sourced from the Mortality Information System of the SUS Information Technology Department, categorized by ICD-9 and ICD-10.

| **Health problem** | **ICD-9** | **ICD-10** |
| --- | --- | --- |
| **Suicide** |  |  |
| Suicide by firearm | E955 | X72 to X74 |
| **Other external causes of accidental injuries** |  |  |
| Other external causes of accidental injuries by hanging, strangulation, and suffocation. | E913 | W75 to W76 |
| Other external causes of accidental injuries by accidental autointoxication. | E850 to E869 | X40 to X49 |
| Other external causes of accidental injuries by firearm | E922 | W32 to W34 |
| **Assault** |  |  |
| Homicide by hanging/strangulation/suffocation | E963 | X91 |
| Homicide by autointoxication | E962 | X85 to X90 |
| Homicide by firearm | E965 | X93 to X95 |
| **Event of undetermined intent** |  |  |
| Hanging/strangulation/suffocation, intent undetermined | E983 | Y20 |
| Intoxication or autointoxication, intent unknown (whether intentional or accidental) | E980 to E982 | Y1 to Y19 |
| Firearm discharge, rifle, pistol, other firearms, intent undetermined | E985 | Y22 to Y24 |
| Sequelae of self-inflicted injury | E959 | Y87 |

Source: 9th and 10th editions of the International Classification of Diseases and Related Health Problems.

5- Provide information about all included data sources and their main characteristics. For each data source used, report reference information or contact name/institution, population represented, data collection method, year(s) of data collection, sex and age range, diagnostic criteria or measurement method, and sample size, as relevant.

R- Information was answered in item 3.

6-Identify and describe any categories of input data that have potentially important biases (eg, based on characteristics listed in item 5).

R-Due to the limitations presented by the information system concerning the poor certification and underreporting of death records, this study conducted the correction of death records for poor certification following the steps described in Supplementary Material 2. Mortality rates without correction and corrected at each stage of the correction process were presented according to period and means of suicide for each of the Brazilian regions (S3 Table). Standardized rates were also calculated using the direct method, with the standard population being the world population proposed by the WHO, in order to correct for the effect of the different age structures of the Brazilian regions during the study period.

***For all data inputs from multiple sources that are synthesised as part of the study:***

7-Describe and give sources for any other data inputs.

R-Relevant study information that was not included in the article was provided through supplementary material.

***For all data inputs:***

8-Provide all data inputs in a file format from which data can be efficiently extracted (eg, a spreadsheet rather than a PDF), including all relevant meta-data listed in item 5. For any data inputs that cannot be shared because of ethical or legal reasons, such as third-party ownership, provide a contact name or the name of the institution that retains the right to the data.

R- Access to the database and R codes used in this study will be provided, through a free and open-access repository Zenodo, along with the accepted version of the manuscript.

Data analysis

9-Provide a conceptual overview of the data analysis method. A diagram may be helpful.

R In the study methodology we describe the source of data acquisition, the variables under study, the process of correcting data records. Once the deaths were corrected, specific rates were estimated by age group, period and cohort using the Epi library in the R program, then the APC models were estimated using estimable functions.

10-Provide a detailed description of all steps of the analysis, including mathematical formulae. This description should cover, as relevant, data cleaning, data pre-processing, data adjustments and weighting of data sources, and mathematical or statistical model(s).

R- The method used was well described in the manuscript, as presented below.

To assess the effect of age, period, and cohort, we grouped age ranges and periods into five-year intervals. Age groups from 10 to 14 years to 80 years or more were chosen due to an excess of zeros in smaller age groups.

The APC effects were estimated using regression models with a Poisson distribution for the number of observed deaths in each age group i and period j (θij). These effects are additively related to the logarithm of the expected mortality rate (E(rij)), following Holford's proposal [30].

$$ln\left( E\left[ r_{ij} \right] \right)=ln\left( \frac{\theta_{ij}}{N_{ij}} \right)=\mu+\alpha_{i}+\beta_{j}+\gamma_{k}+\varepsilon_{ij}$$

Where $E[r_{ij}]$ denotes the expected rate, $\theta_{ij}$ the number of observed deaths, and $N_{ij}$ the population at risk of death in age group $i$ and period $j$. The parameter $\mu$ represents the average effect, $\alpha_{i}$ represents the effect of age group $i$, $\beta_{ij}$ the effect of period $j$, and $\gamma_{k}$the effect of cohort $k (k = 1,...K. K = I + J - 1 = 22)$ and$\varepsilon_{ij}$ represents the random error in age (i) and period (j). Here, i = 1, ..., I; j = 1, ... J; k = 1, ..., K; where K = I + J-1. I correspond to the number of age groups, J to the number of periods, and K to the number of cohorts. Consequently, we obtained I = 15 age groups, J = 8 periods, and K = I + J – 1 = 22 birth cohorts (1900 to 2009).

Estimable functions are limited to the analysis of linear combinations and curvature effects of temporal terms (age, period, and cohort). The linear trend of effects is divided into two components: the linear effect of age and the drift effect (linear effect of period and cohort). The longitudinal trend of age is the sum of age and period slope (αL + βL), where αL and βL are the linear trends of age and period, respectively. The second drift term represents the linear trend of the logarithm of specific rates (mortality) by age and is equal to the sum of period and cohort slopes (βL + γL), where βL and γL are the linear trends of period and cohort, respectively [30,43].

Fifteen APC analyses (scenarios) were conducted for female suicides by firearm, autointoxication, and hanging/strangulation/suffocation in the five Brazilian regions. In each scenario, the adjusted APC submodels were compared in a nested manner via deviance statistics and likelihood ratio tests at a 5% significance level, as proposed by Holford [30,43].

The deviance analysis of the APC models determined by the Epi library estimates six nested equations, namely: (1) f(a) age; (2) f(a) + δc age-drift; (3) f(a) + h(c) age-cohort; (4) f(a) + g(p) + h(c) age-period-cohort; (5) f(a) + g(p) age-period; and (6) f(a) + δp age-drift. Here, a, p, and c represent the effects of age, period, and cohort; f, h, and g are smooth functions of parameters; and δ is a linear effect [30,43].

Based on the best-fit model, estimated specific mortality rates by age and relative risks (RR) were extracted for each period and cohort concerning their respective reference categories (period: 2000-2004; birth cohort: 1950-1954). Interval estimates were obtained at a 95% confidence level [30,43]. We chose the five-year period 2000-2004 as it marks the implementation of significant suicide prevention measures (National Mental Health Policy and Disarmament Statute) [28,44-45]. Regarding the reference cohort, we selected 1950-1954 because median cohorts tend to have a greater quantity of values, being more stable and complete than the first and last ones [30-31,43]. Furthermore, previous studies conducted in Brazil have shown a lower risk of death in generations born from the 1950s onwards [28,44-45].

11- Describe how candidate models were evaluated and how the final model(s) were selected.

R- Estimable functions are limited to the analysis of linear combinations and curvature effects of temporal terms (age, period, and cohort). The linear trend of effects is divided into two components: the linear effect of age and the drift effect (linear effect of period and cohort). The longitudinal trend of age is the sum of age and period slope (αL + βL), where αL and βL are the linear trends of age and period, respectively. The second drift term represents the linear trend of the logarithm of specific rates (mortality) by age and is equal to the sum of period and cohort slopes (βL + γL), where βL and γL are the linear trends of period and cohort, respectively [30,43].

Fifteen APC analyses (scenarios) were conducted for female suicides by firearm, autointoxication, and hanging/strangulation/suffocation in the five Brazilian regions. In each scenario, the adjusted APC submodels were compared in a nested manner via deviance statistics and likelihood ratio tests at a 5% significance level, as proposed by Holford [30,43].

The deviance analysis of the APC models determined by the Epi library estimates six nested equations, namely: (1) f(a) age; (2) f(a) + δc age-drift; (3) f(a) + h(c) age-cohort; (4) f(a) + g(p) + h(c) age-period-cohort; (5) f(a) + g(p) age-period; and (6) f(a) + δp age-drift. Here, a, p, and c represent the effects of age, period, and cohort; f, h, and g are smooth functions of parameters; and δ is a linear effect [30,43].

12- Provide the results of an evaluation of model performance, if done, as well as the results of any relevant sensitivity analysis.

R- The model results were presented in the supplementary material.

13-Describe methods of calculating uncertainty of the estimates. State which sources of uncertainty were, and were not, accounted for in the uncertainty analysis.

R-In all model results, estimates and their respective confidence intervals and p-values ​​were presented (Table 4, Supplementary material S4 Table and S5 Table).

**Results and discussion**

14- Provide published estimates in a file format from which data can be efficiently extracted.

R- All results of the manuscript were presented in a format that allows data extraction

15- Report a quantitative measure of the uncertainty of the estimates (eg, uncertainty intervals).

R- The relative risk and their respective confidence intervals were presented. In the description of the results, we present what was considered statistically significant: Example: increasing risk (RR>1, p<0.05) and lower risk (RR<1, p<0.05) and the detailed values ​​are in supplementary material 6. In the article, we present the results of the APC model in graphs.

16- Interpret results in light of existing evidence. If updating a previous set of estimates, describe the reasons for changes in estimates.

17- Interpret results in light of existing evidence. If updating a previous set of estimates, describe the reasons for changes in estimates.

R- We discuss our results pointing out the similarities and differences with other Brazilian and international studies.

18- Discuss limitations of the estimates. Include a discussion of any modelling assumptions or data limitations that affect interpretation of the estimates.

R-The poor certification and underreporting of death records in SIM are limitations of the present study. However, we employed demographic and epidemiological techniques to minimize this weakness and generate more reliable mortality rates. Another limitation is the inability to perform analyses based on race, sexual orientation, and gender identity, as studies reveal differences in suicidal behavior among women in these different groups [5-6]. These analyses were not conducted because the SIM lacks information on sexual orientation and gender identity, making it impossible to estimate APC models disaggregated by race/color, sexual orientation, and gender identity. Another limitation concerns APC models, as there is no consensus in the literature on the best method to correct the identification problem that occurs in their estimation. This is due to the linear relationship between age, period, and cohort, allowing the derivation of infinite maximum likelihood models with different parameters and distinct estimations. However, they will produce the same prediction for any combination of these factors, hindering the estimation of the complete model [30-31,43]. In this study, we adopted the methodology most frequently recommended by authors who established comparisons between classical statistical methods, namely, estimable functions [30-31]. Furthermore, the high risk of death from hanging/strangulation/suffocation in younger generations must be analyzed with caution, as they may be correlated with the lower number of observations in these cohorts, one of the drawbacks of the method used.
