# Supplementary material for: Suicide methods among Brazilian women from 1980 to 2019: Influence of age, period, and cohort
Source: PLoS One. 2024 Dec 13;19(12):e0311360. doi: 10.1371/journal.pone.0311360 (PMC11642912; doi:10.1371/journal.pone.0311360)
Supplement: S2 Table — (DOCX) [file pone.0311360.s002.docx]

# S2. Stages of the process for correcting suicides by method of perpetration for information quality and coverage of death records.

The death records were initially corrected for information quality through proportional redistribution, according to year, age group, locality, and method of perpetration in six steps according to the method proposed by Garcia et al. (2015).

**(Stage 1)-** The proportion of suicides by each method of perpetration (hanging, strangulation/suffocation, firearm, and self-intoxication) was calculated in relation to the total suicides by year, age group, and locality.

**(Stage 2)-** The results obtained in the previous step were multiplied by the total number of death records classified as "Lon-term consequences of intentionally self-inflicted injuries," obtaining the quantity of this classification to be redistributed by year and age group for suicides according to the method of perpetration (hanging, strangulation/suffocation, firearm, and self-poisoning) by year, age group, and locality;

**(Stage 3)-** The records of suicide, aggression, and other external causes of accidental trauma were aggregated according to the method of perpetration, by year, age group, and locality;

**(Stage 4)-** The deaths by suicide according to the method of perpetration, year, age group, and locality were divided by the results obtained in step 3;

**(Stage 5)-** The values from step 4 were multiplied with the records classified as events with undetermined intent according to the methods of perpetration, year, age group, and locality;

**(Stage 6):-** The values obtained in step 5 were added to the results from step 2 to the total of suicides according to the method of perpetration originally recorded in the SIM, by year, age group, and locality;

**(Stage 7)-** The results from step six were multiplied by the correction factors for underreporting of deaths by year, age group, locality, and method of perpetration.

After the deaths were corrected for incorrect certification of the underlying cause, rectification was performed for underreporting, using the coverage degree of female death records according to geographical region and decade, estimated by demographic methods of distribution of Adjusted Synthetic Extinct Generations (SEG-adj), which consists of a combination of the extinct generations’ method and the general balancing equation (Hill et al., 2009). The estimated coverage degree was provided by researchers from the Laboratory of Population Estimates and Projections at UFRN (LEPP). The process of rectifying the records was carried out by two independent researchers and verified by a third.

# S1.Table Stages of the process for correcting suicides by method of perpetration for information quality and coverage of death records.

|  |
| --- |
| **Suicide by hanging, strangulation, and suffocation.** |
| **ICD-9 and ICD-10 Codes** |
| Suicide by hanging, strangulation, and suffocation (E95 and X70) |
| Total suicides (E950 to E958 and X60 to X84) |
| Other external causes of accidental trauma by hanging, strangulation, and suffocation (E913 and W75 and W76) |
| Assault by hanging, strangulation, and suffocation (E963 and X91). |
| Hanging, strangulation, and suffocation, undetermined intent (E983 and Y20) |
| Long-term consequences of intentionally self-inflicted injuries (E959 and Y87) |
| **Stages of the correction process for information quality through proportional redistribution by year, age group, and locality** |
| Phase 1- Division of E95 and X70 by E950 to E958 and X60 to X84^a^ |
| Phase 2- Multiplication of the results obtained in phase 1 by the total number of deaths for E959 and Y87^b^ |
| Phase 3- Addition of the following codes: E95 and X70 + E913 + W75 + W76 + E963 + X91. |
| Phase 4- Division of the deaths E95 and X70 by the results of phase 3 (E95 and X70 + E913 +W75+W76+ E963+X91). |
| Phase 5- Multiplication of the results of phase 4 by the values of E983 and Y20^c^ |
| Phase 6- Addition of the results obtained in phase 5 to the deaths for E95 and X70, and to the values obtained in phase 2^d^  ^a^Proportion of suicide by hanging, strangulation, and suffocation in the total number of suicides by year and age group; ^b^Identification of the number of deaths classified as E959 and Y87 to redistribute by year and age group for suicide by hanging, strangulation, and suffocation; ^c^Identification of the number of deaths classified as E983 and Y20 to redistribute by year and age group for suicides by hanging, strangulation, and suffocation; ^d^Suicide by hanging, strangulation, and suffocation corrected for information quality by year and age group. |
| **Correction for the quality of information and coverage of deaths** |
| Phase 7- Multiplication of the results from phase 6 by the correction factors for death coverage.  ^e^Suicide by hanging, strangulation, and suffocation corrected for information quality and coverage by year and age group. |
| **Suicide by firearm** |
| **CID-9 and CID-10 Codes** |
| Suicide by firearm (E955 and X72 to X74)  Total suicides (E950 to E958 and X60 to X84)  Other external causes of accidental trauma by firearm (E922 and W32 to W34)  Assault by firearm (E965 and X93 to X95)  Firearm discharge, rifle, pistol, other firearms of undetermined intent (E985 and Y22 to Y24)  Long-term consequences of intentionally self-inflicted injuries (E959 and Y87) |
| **Stages of the information quality correction process through proportional redistribution by year, age group, and locality** |
| Phase 1- Division of E955 and X72 to X74 by E950 to E958 and X60 to X84^f^ |
| Phase 2- Multiplication of the results obtained in phase 1 by the total number of deaths for E959 and Y87^g^ |
| Phase 3- Addition of the following codes: E955 + X72 to X74 + E922 + W32 to W34 + X93 to X95. |
| Phase 4- Division of the deaths for E955 and X72 to X74 by the results of phase 3 (E955 + X72 to X74 + E922 + W32 to W34 + X93 to X95). |
| Phase 5- Multiplication of the outcomes of phase 4 by the values corresponding to E985 and Y22 to Y24^h^ |
| Phase 6- Addition of the outcomes from phase 5 to the deaths for E955 and X72 to X74, and to the results from phase 2^i^  ^f^ Proportion of firearm suicides to the total number of suicides by year and age group; ^g^Identification of the number of deaths classified as E959 and Y87 to redistribute by year and age group for firearm suicide; ^h^Identification of the number of deaths classified as E985 and Y22 to Y24 to redistribute by year and age group for firearm suicides; ^i^Firearm suicide corrected for information quality. |
| **Correction for the quality of information and coverage of deaths** |
| Phase 7- Multiplication of the results from phase 6 by the correction factors for death coverage^j^  ^j^ Firearm suicide corrected for information quality and coverage. |
| **CID-9 e CID-10 Codes** |
| Suicide by self-intoxication (E950 to E952 and X60 to X69) |
| Total suicides (E950 to E958 and X60 to X84) |
| Other external causes of accidental trauma: poisoning and accidental poisoning (E850 to E869 and X40 to X49) |
| Assault by chemicals, drugs, pesticides, gases, and vapors (E962 and X85 to X90) |
| Poisoning and intoxication of undetermined intent (E980 to E982 and Y10 to Y19) |
| Lon-term consequences of intentionally self-inflicted injuries (E959 and Y87) |
| **Phases of the information quality correction process through proportional redistribution by year, age group, and locality** |
| Phase 1- Division of E950 to E958 and X60 to X69 by E950 to E958 and X60 to X84^k^ |
| Phase 2- Multiplication of the value obtained in phase 1 by the total number of deaths for E959 and Y87^l^ |
| Phase 3- Addition of the following codes: E950 to E952 + X60 to X69 + E850 to E869 + X40 to X49 + X85 to X89 |
| Phase 4- Division of deaths for E950 to E952 and X60 to X69 by the values obtained in phase 3 (E950 to E952 + X60 to X69 + E850 to E869 + X40 to X49 + X85 to X89) |
| Phase 5- Multiplication of the results from phase 4 by the values of E980 to E982 and Y10 to Y19^m^ |
| Phase 6- Addition of the results from phase 5 to the deaths for E950 to E958 and X60 to X69, and to the values obtained in phase 2^n^  ^k^ Proportion of suicide by self-poisoning to the total number of suicides by year and age group; ^l^ Identification of the number of deaths classified as E959 and Y87 to redistribute by year and age group for self-poisoning suicide; ^m^ Identification of the number of deaths classified as E980 to E982 and Y10 to Y19 to redistribute by year and age group for self-poisoning suicides; ^n^ Self-intoxication suicides corrected for information quality. |
| **Correction for the quality of information and coverage of deaths** |
| Phase 7- Multiplication of the results of phase 6 by the correction factors for death coverage^o^  ^o^Suicides by self-intoxication corrected for quality of information and coverage. |

References

Garcia LP, Freitas LRS, Silva GDM, Höfelmann DA. Estimativas corrigidas de feminicídios no Brasil, 2009 a 2011. Rev Panam Salud Publica 2015;37(4-5):251-

Hill K, You D, Choi Y. Death Distribution Methods for Estimating Adult Mortality: sensitivity analysis with simulated data errors. Demographic Research. 2009; vol 21, 235:25.

Laboratório de Estimativas e Projeções Populacionais da UFRN (LEPP).https://demografiaufrn.net/laboratorios/lepp/[ acessado em 19 de novembro de 2023).
