# Supplementary material for: Suicide methods among Brazilian women from 1980 to 2019: Influence of age, period, and cohort
Source: PLoS One. 2024 Dec 13;19(12):e0311360. doi: 10.1371/journal.pone.0311360 (PMC11642912; doi:10.1371/journal.pone.0311360)
Supplement: S3 Table — Brazil, 1980–2019. (DOCX) [file pone.0311360.s003.docx]

**S3 Table.** Suicide rates per 100,000 women, by means of perpetration, stages of correction of records, decade, and region. Brazil, 1980-2019.

| **Perpetration method** |  |  |  |  |  |
| --- | --- | --- | --- | --- | --- |
| **North** | **Rate** | **Decade** | | | |
|  |  | **1980-89** | **1990-99** | **2000-09** | **2010-19** |
|  |  | | | | |
| Hanging/strangulation/suffocation | UMR^a^ | 0.52 | 0.61 | 0.98 | 1.80 |
|  | MRCIQ^b^ | 0.54 | 0.65 | 1.07 | 1.90 |
|  | MRCIQC^c^ | 0.85 | 0.88 | 1.44 | 2.55 |
|  | ∆%^d^ | 62.73 | 44.66 | 46.19 | 41.91 |
| Autointoxication | UMR^a^ | 0.50 | 0.54 | 0.59 | 0.39 |
|  | MRCIQ^b^ | 0.65 | 0.71 | 0.68 | 0.44 |
|  | MRCIQC^c^ | 1.22 | 1.18 | 1.16 | 0.62 |
|  | ∆%^d^ | 144.79 | 117.14 | 94.32 | 60.46 |
| Firearm | UMR^a^ | 0.23 | 0.21 | 0.12 | 0.08 |
|  | MRCIQ^b^ | 0.28 | 0.25 | 0.16 | 0.08 |
|  | MRCIQC^c^ | 0.38 | 0.31 | 0.16 | 0.11 |
|  | ∆%^d^ | 66.16 | 46.87 | 36.04 | 35.10 |
| **Northeast** |  | | | | |
| Hanging/strangulation/suffocation | UMR^a^ | 0.32 | 0.53 | 0.85 | 1.21 |
|  | MRCIQ^b^ | 0.39 | 0.58 | 0.91 | 1.29 |
|  | MRCIQC^c^ | 0.54 | 0.70 | 1.17 | 1.66 |
|  | ∆%^d^ | 67.13 | 30.91 | 38.34 | 37.18 |
| Autointoxication | UMR^a^ | 0.24 | 0.29 | 0.66 | 0.64 |
|  | MRCIQ^b^ | 0.67 | 0.68 | 1.48 | 1.28 |
|  | MRCIQC^c^ | 0.93 | 0.81 | 1.90 | 1.64 |
|  | ∆%^d^ | 290.52 | 180.97 | 186.07 | 156.06 |
| Firearm | UMR^a^ | 0.12 | 0.16 | 0.08 | 0.05 |
|  | MRCIQ^b^ | 0.13 | 0.17 | 0.09 | 0.05 |
|  | MRCIQC^c^ | 0.18 | 0.21 | 0.11 | 0.07 |
|  | ∆%^d^ | 54.63 | 29.43 | 39.96 | 30.83 |
| **Southeast** | | | | | |
| Hanging/strangulation/suffocation | UMR^a^ | 0.47 | 0.53 | 0.61 | 1.10 |
|  | MRCIQ^b^ | 0.57 | 0.65 | 0.68 | 1.21 |
|  | MRCIQC^c^ | 0.59 | 0.68 | 0.71 | 1.25 |
|  | **∆**%^d^ | 26.73 | 29.49 | 15.75 | 14.12 |
| Autointoxication | UMR^a^ | 0.55 | 0.34 | 0.55 | 0.58 |
|  | MRCIQ^b^ | 0.96 | 0.60 | 0.78 | 0.73 |
|  | MRCIQC^c^ | 1.00 | 0.63 | 0.81 | 0.76 |
|  | **∆**%^d^ | 82.67 | 87.44 | 46.80 | 30.94 |
| Firearm | UMR^a^ | 0.25 | 0.24 | 0.12 | 0.09 |
|  | MRCIQ^b^ | 0.32 | 0.34 | 0.27 | 0.18 |
|  | MRCIQC^c^ | 0.33 | 0.36 | 0.28 | 0.18 |
|  | **∆**%^d^ | 30.67 | 51.19 | 136.30 | 108.00 |
| **South** | | | | | |
| Hanging/strangulation/suffocation | UMR^a^ | 2.45 | 2.23 | 1.96 | 2.38 |
|  | MRCIQ^b^ | 2.68 | 2.37 | 2.03 | 2.47 |
|  | MRCIQC^c^ | 2.82 | 2.49 | 2.11 | 2.57 |
|  | **∆**%^d^ | 14.79 | 11.23 | 7.70 | 7.93 |
| Autointoxication | UMR^a^ | 1.07 | 0.67 | 0.67 | 0.80 |
|  | MRCIQ^b^ | 1.61 | 0.90 | 0.79 | 0.93 |
|  | MRCIQC^c^ | 1.69 | 0.94 | 0.82 | 0.97 |
|  | **∆**%^d^ | 58.11 | 39.87 | 23.18 | 21.55 |
| Firearm | UMR^a^ | 0.54 | 0.76 | 0.49 | 0.26 |
|  | MRCIQ^b^ | 0.75 | 0.89 | 0.51 | 0.26 |
|  | MRCIQC^c^ | 0.78 | 0.94 | 0.53 | 0.27 |
|  | **∆**%^d^ | 44.96 | 22.70 | 8.50 | 5.73 |
| **Midwest** | | | | | |
| Hanging/strangulation/suffocation | UMR^a^ | 0.50 | 0.86 | 1.48 | 1.91 |
|  | MRCIQ^b^ | 0.58 | 0.93 | 1.53 | 1.97 |
|  | MRCIQC^c^ | 0.72 | 1.01 | 1.67 | 2.15 |
|  | **∆**%^d^ | 43.88 | 17.48 | 12.72 | 12.60 |
| Autointoxication | UMR^a^ | 1.22 | 0.99 | 1.06 | 0.83 |
|  | MRCIQ^b^ | 1.91 | 1.36 | 1.34 | 1.08 |
|  | MRCIQC^c^ | 2.37 | 1.48 | 1.45 | 1.17 |
|  | **∆**%^d^ | 93.65 | 49.82 | 37.63 | 41.27 |
| Firearm | UMR^a^ | 0.40 | 0.57 | 0.34 | 0.18 |
|  | MRCIQ^b^ | 0.53 | 0.65 | 0.35 | 0.18 |
|  | MRCIQC^c^ | 0.66 | 0.71 | 0.38 | 0.19 |
|  | **∆**%^d^ | 67.16 | 23.38 | 10.61 | 9.74 |

Note: ^a^Standardized mortality rates without correction; ^b^Standardized mortality rates after correction for poor certification of deaths; ^c^Standardized mortality rates after correction for poor certification of deaths and coverage; ^d^Percentage change in standardized mortality rates after correction for poor certification of deaths and coverage compared to standardized mortality rates without correction.
