# Supplementary material for: Suicide methods among Brazilian women from 1980 to 2019: Influence of age, period, and cohort
Source: PLoS One. 2024 Dec 13;19(12):e0311360. doi: 10.1371/journal.pone.0311360 (PMC11642912; doi:10.1371/journal.pone.0311360)
Supplement: S4 Table — (DOCX) [file pone.0311360.s004.docx]

**S4 Table**.Deviance, Akaike Information Criterion (AIC) an p-value analysis in sequential construction of age, period, and cohort models for suicides in women by methods, according to Brazil its major regions, from 1980 to 2019.

| **Models** | **DF^a^** | ***Deviance* residual** | ***AIC*** | **p (>\|Chi\|)** |
| --- | --- | --- | --- | --- |
| **North** |  |  |  |  |
| **Hanging, strangulation and suffocation** | | | | |
| Age | 115 | 977.92 | 1500.10 |  |
| Age-drift^b^ | 114 | 292.65 | 816.83 | <0.0001 |
| Age-Cohort | 111 | 230.54 | 760.72 | <0.0001 |
| Age-Period-Cohort | 109 | 221.15 | 755.33 | <0.0001 |
| Age-Period | 112 | 263.51 | 791.70 | <0.0001 |
| Age-drift^c^ | 114 | 292.65 | 816.83 | <0.0001 |
| **Autointoxication** | | | | |
| Age | 115 | 931.27 | 516.63 |  |
| Age-drift^b^ | 114 | 852.20 | 435.56 | <0.0001 |
| Age-Cohort | 111 | 824.06 | 401.42 | <0.0001 |
| Age-Period-Cohort | 109 | 799.40 | 370.76 | <0.0001 |
| Age-Period | 112 | 823.48 | 400.84 | <0.0001 |
| Age-drift^c^ | 114 | 852.20 | 435.56 | <0.0001 |
| **Firearm** | | | | |
| Age | 115 | 512.17 | 261.54 |  |
| Age-drift^b^ | 114 | 400.97 | 148.34 | <0.0001 |
| Age-Cohort | 111 | 401.37 | 142.75 | <0.0001 |
| Age-Period-Cohort | 109 | 399.63 | 135.00 | <0.0001 |
| Age-Period | 112 | 397.44 | 138.81 | <0.0001 |
| Age-drift^c^ | 114 | 400.97 | 148.34 | <0.0001 |
| **Northeast** | | | | |
| **Hanging, strangulation and suffocation** | **DF** | **Deviance residual** | **AIC** | **p (>\|Chi\|)** |
| Age | 115 | 2807.17 | 2121.96 |  |
| Age-drift^b^ | 114 | 1356.76 | 669.54 | <0.0001 |
| Age-Cohort | 111 | 1207.55 | 514.34 | <0.0001 |
| Age-Period-Cohort | 108 | 1189.51 | 490.29 | <0.0001 |
| Age-Period | 111 | 1339.19 | 645.97 | <0.0001 |
| Age-drift^c^ | 114 | 1356.76 | 669.54 | <0.0001 |
| **Autointoxication** | | | | |
| Age | 115 | 2300.08 | 1684.26 |  |
| Age-drift^b^ | 114 | 1684.19 | 1066.37 | <0.0001 |
| Age-Cohort | 111 | 1608.71 | 984.89 | <0.0001 |
| Age-Period-Cohort | 108 | 1261.46 | 631.64 | <0.0001 |
| Age-Period | 111 | 1298.42 | 674.60 | <0.0001 |
| Age-drift^c^ | 114 | 1684.19 | 1066.37 | <0.0001 |
| **Firearm** | | | | |
| Age | 115 | 807.30 | 428.95 |  |
| Age-drift^b^ | 114 | 645.66 | 265.32 | <0.0001 |
| Age-Cohort | 111 | 650.74 | 264.39 | <0.0001 |
| Age-Period-Cohort | 108 | 622.21 | 229.87 | <0.0001 |
| Age-Period | 111 | 619.62 | 233.27 | <0.0001 |
| Age-drift^c^ | 114 | 645.66 | 265.32 | <0.0001 |
| **Southeast** | | | | |
| **Hanging, strangulation and suffocation** | **DF** | **Deviance residual** | **AIC** | **p (>\|Chi\|)** |
| Age | 115 | 2470.31 | 1755.85 |  |
| Age-drift^b^ | 114 | 1517.89 | 801.43 | <0.0001 |
| Age-Cohort | 111 | 1255.89 | 533.43 | <0.0001 |
| Age-Period-Cohort | 108 | 1138.07 | 409.61 | <0.0001 |
| Age-Period | 111 | 1302.25 | 579.79 | <0.0001 |
| Age-drift^c^ | 114 | 1517.89 | 801.43 | <0.0001 |
| **Autointoxication** | | | | |
| Age | 115 | 2052.48 | 1339.59 |  |
| Age-drift^b^ | 114 | 1986.99 | 1272.10 | <0.0001 |
| Age-Cohort | 111 | 1688.59 | 967.70 | <0.0001 |
| Age-Period-Cohort | 108 | 1288.65 | 561.76 | <0.0001 |
| Age-Period | 111 | 1653.48 | 932.59 | <0.0001 |
| Age-drift^c^ | 114 | 1986.99 | 1272.10 | <0.0001 |
| **Firearm** | | | | |
| Age | 115 | 1223.71 | 648.89 |  |
| Age-drift^b^ | 114 | 1062.53 | 485.71 | <0.0001 |
| Age-Cohort | 111 | 1032.56 | 449.74 | <0.0001 |
| Age-Period-Cohort | 108 | 977.91 | 389.09 | <0.0001 |
| Age-Period | 111 | 989.69 | 406.87 | <0.0001 |
| Age-drift^c^ | 114 | 1062.53 | 485.71 | <0.0001 |
| **South** | | | | |
| **Hanging, strangulation and suffocation** | **DF** | **Deviance residual** | **AIC** | **p (>\|Chi\|)** |
| Age | 115 | 1378.93 | 635.98 |  |
| Age-drift^b^ | 114 | 1376.49 | 631.55 | <0.0001 |
| Age-Cohort | 111 | 1140.51 | 389.56 | <0.0001 |
| Age-Period-Cohort | 108 | 1100.06 | 343.11 | <0.0001 |
| Age-Period | 111 | 1304.28 | 343.11 | <0.0001 |
| Age-drift^c^ | 114 | 1376.49 | 631.55 | <0.0001 |
| **Autointoxication** | | | | |
| Age | 115 | 1588.94 | 977.93 |  |
| Age-drift^b^ | 114 | 1400.50 | 787.48 | <0.0001 |
| Age-Cohort | 111 | 1281.14 | 662.13 | <0.0001 |
| Age-Period-Cohort | 108 | 969.68 | 344.66 | <0.0001 |
| Age-Period | 111 | 1149.72 | 530.71 | <0.0001 |
| Age-drift^c^ | 114 | 1400.50 | 787.48 | <0.0001 |
| **Firearm** | | | | |
| Age | 115 | 1291.51 | 755.45 |  |
| Age-drift^b^ | 114 | 934.03 | 395.97 | <0.0001 |
| Age-Cohort | 111 | 866.70 | 322.64 | <0.0001 |
| Age-Period-Cohort | 108 | 772.23 | 222.16 | <0.0001 |
| Age-Period | 111 | 798.23 | 254.17 | <0.0001 |
| Age-drift^c^ | 114 | 934.03 | 395.97 | <0.0001 |
| **Midwest** | | | | |
| **Hanging, strangulation and suffocation** | **DF** | **Deviance residual** | **AIC** | **p (>\|Chi\|)** |
| Age | 115 | 1350.06 | 828.25 |  |
| Age-drift^b^ | 114 | 872.76 | 348.95 | <0.0001 |
| Age-Cohort | 111 | 819.42 | 289.61 | <0.0001 |
| Age-Period-Cohort | 108 | 789.11 | 255.30 | <0.0001 |
| Age-Period | 111 | 855.92 | 328.11 | <0.0001 |
| Age-drift^c^ | 114 | 872.76 | 348.95 | <0.0001 |
| **Autointoxication** | | | | |
| Age | 115 | 1142.38 | 613.92 |  |
| Age-drift^b^ | 114 | 946.31 | 415.85 | <0.0001 |
| Age-Cohort | 111 | 910.88 | 374.42 | <0.0001 |
| Age-Period-Cohort | 108 | 898.44 | 355.98 | <0.0001 |
| Age-Period | 111 | 933.19 | 396.73 | <0.0001 |
| Age-drift^c^ | 114 | 946.31 | 415.85 | <0.0001 |
| **Firearm** | | | | |
| Age | 115 | 795.86 | 436.41 |  |
| Age-drift^b^ | 114 | 604.58 | 243.13 | <0.0001 |
| Age-Cohort | 111 | 597.35 | 229.90 | <0.0001 |
| Age-Period-Cohort | 108 | 560.18 | 186.73 | <0.0001 |
| Age-Period | 111 | 563.61 | 196.16 | <0.0001 |
| Age-drift^c^ | 114 | 604.58 | 243.13 | <0.0001 |

Note:^a^Degrees of freedom^; b^represents the linear trend of the logarithm of age throughout time, and is equal to the sum of the slopes of period and cohort $\left( \text{β}_{\text{L}}\text{+}\text{γ}_{\text{L}} \right)$, where $\text{β}_{\text{L}}$ and $\gamma_{L}$ are the linear trends for the period and cohort, respectively; ^c^represents longitudinal trend of age, equal to the sum of age and period slope (αL + βL), being αL and βL linear trends of age and period, respectively
