# Supplementary material for: Suicide methods among Brazilian women from 1980 to 2019: Influence of age, period, and cohort
Source: PLoS One. 2024 Dec 13;19(12):e0311360. doi: 10.1371/journal.pone.0311360 (PMC11642912; doi:10.1371/journal.pone.0311360)
Supplement: S5 Table — (DOCX) [file pone.0311360.s005.docx]

S5 Table - Relative risk and 95% confidence interval for the effect of period and cohort for suicides in women, according to method and geographic region, Brazil 1980 to 2019.

| **Hanging, strangulation and suffocation** | | | | | |
| --- | --- | --- | --- | --- | --- |
| **Period** | **Means-Relative Risk (CI-95%)** | | | | |
|  | **North** | **Northeast** | **Southeast** | **South** | **Midwest** |
| 1980 to 1984 | **1.20( 1.05-1.36)*** | 1.04( 0.96- 1.13) | **1.21( 1.13- 1.29)*** | **1.11( 1.04- 1.18)*** | **0.66 ( 0.57- 0.76)*** |
| 1985 to 1989 | **1.13( 1.04- 1.23)*** | 1.003( 0.95- 1.05) | **1.16( 1.11- 1.20)*** | **1.14( 1.08- 1.21)*** | **0.76 ( 0.70- 0.84)*** |
| 1990 to 1994 | **1.07( 1.02- 1.12)*** | **0.96( 0.93- 0.98)*** | **1.11( 1.07- 1.14)*** | **1.17( 1.09- 1.25)*** | **0.88 ( 0.84- 0.92)*** |
| 1995 to 1999 | 1.02( 1.00- 1.03) | **0.93( 0.91- 0.96)*** | **1.06( 1.02- 1.09)*** | **1.11( 1.06- 1.17)*** | 1.00 ( 0.98- 1.01) |
| 2000 to 2004 | **Reference** | | | | |
| 2005 to 2009 | 1.04 (0.99- 1.09) | **1.13( 1.06- 1.19)*** | 0.96( 0.92* 1.00) | 1.01 ( 0.96- 1.04) | **0.83 ( 0.77- 0.89)*** |
| 2010 to 2014 | **1.14( 1.01- 1.29)*** | 0.99( 0.92- 1.06) | **1.09( 1.02- 1.16)*** | **1.11( 1.03- 1.19)*** | **0.70 ( 0.62- 0.79)*** |
| 2015 to 2019 | **1.21 ( 1.07-1.37)*** | 0.97(0.90- 1.04) | **1.35( 1.28- 1.44)*** | **1.23 ( 1.15- 1.31)*** | **0.72 ( 0.63- 0.82)*** |
| **Autointoxication** | | | | | |
| **Period** | **Means-Relative Risk (CI-95%)** | | | | |
|  | **North** | **Northeast** | **Southeast** | **South** | **Midwest** |
| 1980 to 1984 | **0.71 ( 0.62- 0.83)*** | **1.18( 1.08- 1.28)*** | **1.60( 1.51- 1.69)*** | **2.03 ( 1.88- 2.20)*** | 1.10 ( 0.99- 1.22) |
| 1985 to 1989 | **0.68( 0.60- 0.78)*** | 0.95( 0.91-1.004) | **1.06 (1.01-1.28)*** | **1.39 ( 1.27- 1.53)*** | 0.89 ( 0.80- 1.00) |
| 1990 to 1994 | **0.67 ( 0.57- 0.78)*** | **0.79( 0.76- 0.81)*** | **0.82( 0.77- 0.87)*** | **1.08 ( 1.01- 1.15)*** | **0.81 ( 0.72- 0.92)*** |
| 1995 to 1999 | **0.77( 0.69- 0.87)*** | **0.76 (0.73- 0.78)*** | **0.82(0.80- 0.85)*** | 0.96 ( 0.92- 1.01) | **0.89 ( 0.85- 0.95)*** |
| 2000 to 2004 | **Reference** | | | | |
| 2005 to 2009 | **0.77( 0.67- 0.88)*** | **1.50( 1.42-1.58)*** | **1.24 (1.20 -1.29)*** | **1.15 ( 1.09- 1.22)*** | 0.97 ( 0.89- 1.07) |
| 2010 to 2014 | **0.66( 0.56- 0.77)*** | **1.08( 1.01- 1.17)*** | **1.33( 1.25- 1.43)*** | **1.45( 1.36- 1.55)*** | 0.95 ( 0.85- 1.06) |
| 2015 to 2019 | **0.67( 0.56- 0.78)*** | **0.78( 0.72- 0.85)*** | **1.10( 1.03- 1.18)*** | **1.91( 1.74- 2.10)*** | 0.95 ( 0.84- 1.07) |
| **Firearm** | | | | | |
| **Period** | **Means-Relative Risk (CI-95%)** | | | | |
|  | **North** | **Northeast** | **Southeast** | **South** | **Midwest** |
| 1980 to 1984 | 0.86( 0.63- 1.17) | **0.70( 0.58- 0.86)*** | **0.75 ( 0.68- 0.83)*** | **0.68( 0.60- 0.77)*** | **0.66 ( 0.53- 0.82)*** |
| 1985 to 1989 | 1.17( 0.89- 1.54) | **0.79( 0.67- 0.94)*** | **0.73( 0.66- 0.80)*** | **0.71 ( 0.64- 0.79)*** | **0.82 (0.68- 0.99)*** |
| 1990 to 1994 | **1.35(1.03- 1.78)*** | 1.01( 0.84 -1.19) | **0.82( 0.75- 0.90)*** | 0.90 ( 0.80- 1.00)* | 1.10 ( 0.91- 1.33) |
| 1995 to 1999 | 1.20( 0.93- 1.54) | **1.30(1.11- 1.52)*** | **1.05( 1.01- 1.11)*** | **0.75( 0.69- 0.81)*** | **1.40 ( 1.18- 1.66)*** |
| 2000 to 2004 | **Reference** | | | | |
| 2005 to 2009 | 0.96( 0.86- 1.07) | **0.82( 0.77- 0.88)*** | **0.81(0.76- 0.86)*** | **0.81 (0.78-0.84)*** | **0.80 ( 0.75- 0.86)*** |
| 2010 to 2014 | 1.03( 0.84- 1.26) | **0.80( 0.70- 0.91)*** | **0.74( 0.69- 0.80)*** | **0.75 (0.69 -0.81)*** | **0.79 ( 0.69- 0.91)*** |
| 2015 to 2019 | 1.12( 0.77- 1.64) | 0.79 ( 0.62- 1.01) | **0.75( 0.67- 0.84)*** | **0.71( 0.61- 0.83)*** | 0.80 ( 0.61- 1.05) |
| **Hanging, strangulation and suffocation** | | | | | |
| **Cohort** | **Relative Risk (CI-95%)** | | | | |
|  | **North** | **Northeast** | **Southeast** | **South** | **Midwest** |
| 1900 to 1904 | **0.26( 0.17- 0.40)*** | **0.22( 0.17- 0.28)*** | **0.70(0.57- 0.85)*** | **3.25( 2.75- 3.83)*** | **0.30( 0.19- 0.47)*** |
| 1905 to 1909 | **0.30 ( 0.20- 0.44)*** | **0.26( 0.21- 0.32)*** | **0.72( 0.60- 0.86)*** | **2.80( 2.42- 3.24)*** | **0.34( 0.23- 0.50)*** |
| 1910 to 1914 | **0.34( 0.24- 0.48)*** | **0.35( 0.30- 0.41)*** | **0.75( 0.64- 0.88)*** | **2.42( 2.13- 2.73)*** | **0.38( 0.27- 0.54)*** |
| 1915 to 1919 | **0.39( 0.29- 0.52)*** | **0.40( 0.35- 0.46)*** | **0.77( 0.67- 0.89)*** | **2.08( 1.88- 2.31)*** | **0.43( 0.32- 0.58)*** |
| 1920 to 1924 | **0.44( 0.34- 0.57)*** | **0.47( 0.42- 0.52)*** | **0.80( 0.71- 0.90)*** | **1.80( 1.65- 1.96)*** | **0.48( 0.37- 0.63)*** |
| 1925 to 1929 | **0.50( 0.40- 0.62)*** | **0.47( 0.42- 0.52)*** | **0.82( 0.75- 0.91)*** | **1.55( 1.45- 1.66)*** | **0.55( 0.44- 0.68)*** |
| 1930 to 1934 | **0.57 (0.47- 0.68)*** | **0.54( 0.50- 0.59)*** | **0.85( 0.79- 0.92)*** | **1.34 ( 1.27- 1.41)*** | **0.62( 0.52- 0.73)*** |
| 1935 to 1934 | **0.64 ( 0.56- 0.74)*** | **0.63( 0.59- 0.67)*** | **0.88( 0.83- 0.93)*** | **1.18 ( 1.12- 1.23)*** | **0.69( 0.61- 0.79)*** |
| 1940 to 1944 | **0.73( 0.66- 0.81)*** | **0.73( 0.70 0.76)*** | **0.91( 0.88- 0.94)*** | **1.06 ( 1.02-1.10)*** | **0.78( 0.72- 0.85)*** |
| 1945 to 1949 | **0.83 ( 0.79- 0.88)*** | **0.85( 0.83- 0.86)*** | **0.94( 0.93- 0.96)*** | 1.00( 0.98- 1.03) |  |
| 1950 to 1954 | **Reference** | | | | |
| 1955 to 1959 | **1.08( 1.05- 1.10)*** | **1.18( 1.15- 1.21)*** | **1.09( 1.07- 1.11)*** | **1.02( 1.01- 1.05)*** | **1.13( 1.09- 1.17)*** |
| 1960 to 1964 | **1.24 ( 1.17- 1.31)*** | **1.42( 1.34- 1.50)*** | **1.25( 1.20- 1.31)*** | **1.05( 1.01- 1.10)*** | **1.31( 1.23- 1.40)*** |
| 1965 to 1969 | **1.45( 1.33- 1.57)*** | **1.69( 1.55- 1.83)*** | **1.44( 1.34- 1.54)*** | **1.08( 1.02- 1.13)*** | **1.55( 1.41- 1.71)*** |
| 1970 to 1974 | **1.75 ( 1.57- 1.94)*** | **1.97( 1.79- 2.16)*** | **1.59( 1.48- 1.71)*** | **1.10( 1.03- 1.18)*** | **1.91( 1.66- 2.19)*** |
| 1975 to1979 | **2.18 ( 1.90- 2.50)*** | **2.33( 2.11- 2.56)*** | **1.76( 1.63- 1.89)*** | **1.15( 1.06- 1.24)*** | **2.49( 2.09- 2.96)*** |
| 1980 to 1984 | **2.72( 2.31- 3.20)*** | **2.92( 2.63- 3.24)*** | **2.03( 1.88- 2.20)*** | **1.21( 1.12- 1.31)*** | **3.49( 2.91- 4.17)*** |
| 1985 to 1989 | **3.34 ( 2.83- 3.94)*** | **4.01( 3.58- 4.49)*** | **2.55( 2.34- 2.77)*** | **1.29( 1.18- 1.40)*** | **5.04( 4.14- 6.13)*** |
| 1990 to 1994 | **4.28 ( 3.58- 5.11)*** | **5.90( 5.21- 6.68)*** | **3.35( 3.05- 3.68)*** | **1.37( 1.24- 1.51)*** | **7.19( 5.81- 8.90)*** |
| 1995 to 1999 | **6.00 (4.95-7.28)*** | **8.99( 7.77- 10.40)*** | **4.43( 3.95- 4.97)*** | **1.46( 1.30- 1.64)*** | **10.14( 7.97- 12.89)*** |
| 2000 to 2004 | **8.92 ( 7.08- 11.23)*** | **13.77( 11.55- 16.43)*** | **5.86( 5.10- 6.75)*** | **1.55( 1.36- 1.78)*** | **14.26( 10.71- 18.99)*** |
| 2005 to 2009 | **13.38 ( 9.96- 17.97)*** | **21.10( 17.09- 23.03)*** | **7.76 ( 6.55- 9.19)*** | **1.66( 1.41- 1.94)*** | **20.07( 14.22- 28.31)*** |
| **Autointoxication** | | | | | |
| **Cohort** | **Relative Risk (CI-95%)** | | | | |
|  | **North** | **Northeast** | **Southeast** | **South** | **Midwest** |
| 1900 to 1904 | 0.61 ( 0.32- 1.15) | **0.11 ( 0.08- 0.14)*** | 0.86( 0.70- 1.07) | **1.54( 1.13- 2.11)*** | 1.52( 0.99- 2.34) |
| 1905 to 1909 | 0.64 ( 0.36- 1.13) | **0.13( 0.10- 0.18)*** | 0.87( 0.72- 1.05) | **1.47( 1.11- 1.95)*** | 1.46( 0.99- 2.15) |
| 1910 to 1914 | 0.67( 0.40- 1.12) | **0.17( 0.13- 0.21)*** | 0.88 ( 0.74- 1.04) | **1.41( 1.10- 1.80)*** | 1.40( 0.99- 1.97) |
| 1915 to 1919 | 0.70( 0.44- 1.10) | **0.21 ( 0.17- 0.26)*** | 0.89 (0.77- 1.03) | **1.34( 1.08- 1.67)*** | 1.34 (0.99- 1.81) |
| 1920 to 1924 | 0.73( 0.49- 1.09) | **0.26 ( 0.22- 0.32)*** | 0.90( 0.80- 1.02) | **1.28( 1.07- 1.54)*** | 1.28 (0.99- 1.66) |
| 1925 to 1929 | 0.77( 0.55- 1.07) | **0.33 (0.29- 0.38)*** | 0.91( 0.82- 1.01) | **1.23( 1.05- 1.42)*** | 1.23( 0.99- 1.52) |
| 1930 to 1934 | 0.81( 0.62- 1.06) | **0.41 ( 0.37- 0.46)*** | 0.92 ( 0.85- 1.00) | **1.17( 1.04- 1.32)*** | 1.18( 0.99- 1.40) |
| 1935 to 1934 | 0.85( 0.69- 1.04) | **0.51 ( 0.47- 0.56)*** | **0.93( 0.88- 0.99)*** | **1.12( 1.03- 1.22)*** | 1.13( 0.99- 1.28) |
| 1940 to 1944 | 0.89( 0.77- 1.03) | **0.64( 0.60- 0.68)*** | **0.94( 0.91- 0.98)*** | **1.07( 1.01- 1.13)*** | **1.08( 1.01- 1.18)*** |
| 1945 to 1949 | 0.93( 0.85- 1.01) | **0.80( 0.78- 0.82)*** | **0.96( 0.94- 0.97)*** | **1.02( 1.001- 1.05)*** | **1.04( 1.01- 1.087)*** |
| 1950 to 1954 | **Reference** | | | | |
| 1955 to 1959 | 1.02( 0.99- 1.06) | **1.24( 1.20- 1.27)*** | **1.08 ( 1.06- 1.10)*** | 1.00( 0.97-1.03) | **0.95( 0.92- 0.98)*** |
| 1960 to 1964 | 1.05( 0.96- 1.14) | **1.51 ( 1.44- 1.59)*** | **1.22( 1.16- 1.29)*** | 1.04( 0.96- 1.13) | **0.91( 0.86-0.96)*** |
| 1965 to 1969 | 1.00( 0.89- 1.12) | **1.79 ( 1.68- 1.91)*** | **1.29( 1.20- 1.38)*** | 1.07( 0.96- 1.19) | **0.85( 0.76- 0.95)*** |
| 1970 to 1974 | 0.87( 0.74- 1.02) | **2.05 (1.88- 2.24)*** | **1.16( 1.09- 1.24)*** | 1.03( 0.93- 1.13) | **0.72 ( 0.63- 0.83)*** |
| 1975 to1979 | **0.73( 0.60- 0.88)*** | **2.36 ( 2.12- 2.62)*** | 1.00( 0.93- 1.07) | **0.89( 0.80- 0.98)*** | **0.55( 0.48- 0.62)*** |
| 1980 to 1984 | **0.63 (0.52- 0.75)*** | **2.78( 2.51- 3.08)*** | **0.84 ( 0.79- 0.91)*** | **0.71( 0.64- 0.78)*** | **0.44( 0.39- 0.51)*** |
| 1985 to 1989 | **0.55( 0.46- 0.66)*** | **3.20 (2.88- 3.57)*** | **0.71 ( 0.66- 0.76)*** | **0.55( 0.49- 0.61)*** | **0.39( 0.35- 0.45)*** |
| 1990 to 1994 | **0.48( 0.40- 0.58)***** | **3.43( 3.07- 3.83)*** | **0.59( 0.54- 0.65)*** | **0.42( 0.37- 0.47)*** | **0.37( 0.32- 0.42)*** |
| 1995 to 1999 | **0.43( 0.35- 0.53)*** | **3.61( 3.18- 4.10)*** | **0.50 ( 0.45-0.55)*** | **0.32( 0.27- 0.37)*** | **0.34( 0.29- 0.40)** |
| 2000 to 2004 | **0.38( 0.29- 0.50)*** | **3.80( 3.23- 4.46)*** | **0.42( 0.36- 0.47)*** | **0.24( 0.20- 0.29)*** | **0.32( 0.26- 0.39)*** |
| 2005 to 2009 | **0.34( 0.24- 0.47)*** | **4.00( 3.27- 4.89)*** | **0.35( 0.30- 0.41)*** | **0.18( 0.15- 0.23)*** | **0.30( 0.23- 0.38)*** |
| **Firearm** | | | | | |
| **Cohort** | **Relative Risk (CI-95%)** | | | | |
|  | **North** | **Northeast** | **Southeast** | **South** | **Midwest** |
| 1900 to 1904 | **4.07( 1.03- 16.03)*** | **5.66( 2.51- 12.76)*** | **2.78( 1.85- 4.17)*** | **3.31( 2.04- 5.39)*** | **2.65(1.04- 6.71)*** |
| 1905 to 1909 | **3.53( 1.03- 12.14)*** | **4.76( 2.29- 9.88)*** | **2.50( 1.74- 3.60)*** | **2.95( 1.90- 4.56)*** | **2.40( 1.04- 5.54)*** |
| 1910 to 1914 | **3.07 ( 1.02- 9.20)*** | **4.00( 2.09- 7.66)*** | **2.26( 1.63-3.11)*** | **2.62( 1.78- 3.85)*** | **2.18( 1.03- 4.58)*** |
| 1915 to 1919 | **2.67( 1.02- 6.97)*** | **3.36 (1.91- 5.94)*** | **2.03( 1.54- 2.69)*** | **2.33( 1.66- 3.26)*** | **1.98( 1.03- 3.79)*** |
| 1920 to 1924 | **2.32 (1.02- 5.28)*** | **2.83( 1.74- 4.60)*** | **1.83( 1.44-2.32)*** | **2.07( 1.56- 2.75)*** | **1.79 (1.03- 3.13)*** |
| 1925 to 1929 | **2.01 ( 1.01- 4.00)*** | **2.38( 1.59- 3.56)*** | **1.65( 1.36-2.01)*** | **1.84( 1.45- 2.33)*** | **1.63( 1.02- 2.59)*** |
| 1930 to 1934 | **1.75( 1.01- 3.03)*** | **2.00 ( 1.45- 2.76)*** | **1.49( 1.28- 1.74)*** | **1.64( 1.36- 1.97)*** | **1.48( 1.02- 2.14)*** |
| 1935 to 1934 | **1.52( 1.01- 2.29)*** | **1.68 (1.32- 2.14)*** | **1.34( 1.20- 1.50)*** | **1.45( 1.27- 1.66)*** | **1.34( 1.02- 1.77)*** |
| 1940 to 1944 | **1.32( 1.01- 1.74)*** | **1.41( 1.20- 1.66)*** | **1.21( 1.13- 1.30)*** | **1.29( 1.19- 1.41)*** | **1.22( 1.01-1.46)*** |
| 1945 to 1949 | **1.15( 1.01- 1.31)*** | **1.19( 1.10- 1.28)*** | **1.09( 1.06- 1.13)*** | **1.15( 1.10- 1.19)*** | **1.10( 1.01- 1.21)*** |
| 1950 to 1954 | **Reference** | | | | |
| 1955 to 1959 | **0.86 ( 0.75- 0.98)*** | **0.83 (0.78- 0.88)*** | **0.93( 0.90- 0.96)*** | **0.83(0.80- 0.87)*** | **0.86( 0.81- 0.93)*** |
| 1960 to 1964 | **0.72 ( 0.57- 0.89)*** | **0.68( 0.60- 0.76)*** | **0.89( 0.81- 0.96)*** | **0.67( 0.60- 0.74)*** | **0.70( 0.61- 0.79)*** |
| 1965 to 1969 | **0.56( 0.42- 0.74)*** | **0.54( 0.44- 0.66)*** | **0.85( 0.75- 0.96)*** | **0.56( 0.48- 0.65)*** | **0.52( 0.41- 0.65)*** |
| 1970 to 1974 | **0.42( 0.28- 0.61)*** | **0.42( 0.33- 0.54)*** | **0.80( 0.71- 0.91)*** | **0.52(0.44- 0.60)*** | **0.38( 0.29- 0.50)*** |
| 1975 to1979 | **0.36( 0.24- 0.52)*** | **0.33( 0.26- 0.42)*** | **0.73(0.64- 0.82)*** | **0.46( 0.40- 0.55)*** | **0.29( 0.22- 0.38)*** |
| 1980 to 1984 | **0.31( 0.21- 0.45)*** | **0.28( 0.22- 0.35)*** | **0.63( 0.56- 0.71)*** | **0.37( 0.31- 0.43)*** | **0.23( 0.18- 0.31)*** |
| 1985 to 1989 | **0.22( 0.15- 0.32)*** | **0.24( 0.19- 0.31)*** | **0.53( 0.46- 0.60)*** | **0.26( 0.22- 0.31)*** | **0.18( 0.14- 0.24)*** |
| 1990 to 1994 | **0.15( 0.10- 0.23)*** | **0.22( 0.17- 0.28)*** | **0.44( 0.38- 0.52)*** | **0.18( 0.15- 0.22)*** | **0.14( 0.11- 0.19)*** |
| 1995 to 1999 | **0.11( 0.09- 0.176)*** | **0.19( 0.14- 0.26)*** | **0.37( 0.31- 0.45)*** | **0.13( 0.10- 0.16)*** | **0.11( 0.08- 0.16)*** |
| 2000 to 2004 | **0.08( 0.04- 0.14)*** | **0.17( 0.12- 0.25)*** | **0.31( 0.25- 0.39)*** | **0.09( 0.07- 0.12)*** | **0.09 ( 0.06- 0.14)*** |
| 2005 to 2009 | **0.05( 0.03- 0.10)*** | **0.15( 0.09- 0.24)*** | **0.26( 0.20- 0.34)*** | **0.06( 0.04- 0.09)*** | **0.07( 0.04- 0.12)*** |

*p<0.001
