# Supplementary material for: Suicide methods among Brazilian women from 1980 to 2019: Influence of age, period, and cohort
Source: PLoS One. 2024 Dec 13;19(12):e0311360. doi: 10.1371/journal.pone.0311360 (PMC11642912; doi:10.1371/journal.pone.0311360)
Supplement: Attachment — Submitted filename: Response to reviwers.docx [file pone.0311360.s006.docx]

PONE-D-24-08405

Suicide Methods Among Brazilian Women from 1980 to 2019: Influence of Age, Period, and Cohort

PLOS ONE

Dear Dr. Meira,

Thank you for submitting your manuscript to PLOS ONE. After careful consideration, we feel that it has merit but does not fully meet PLOS ONE’s publication criteria as it currently stands. Therefore, we invite you to submit a revised version of the manuscript that addresses the points raised during the review process.

==============================
**ACADEMIC EDITOR:**

I regret to inform you that we are unable to accept your manuscript for publication in its current form. The reviewers have provided valuable feedback on your manuscript, with two out of three reviews being positive. 
However, the negative review raises significant concerns about the research methodology and the results. 
Therefore, we recommend that you thoroughly address all the comments provided by the reviewers and revise your manuscript accordingly.

**Cite all versions?** You can cite all versions by using the DOI [10.5281/zenodo.13172957](https://zenodo.org/doi/10.5281/zenodo.13172957).

**Response:**

Dear Editor,

We extend our gratitude for the excellent contributions of the reviewers and for the opportunity to revise our manuscript. In the Revised Manuscript with track changes, sentences highlighted in yellow have been removed or replaced by sentences in green. Some sentences that were reordered have been marked in blue. We have organized the responses to the reviewers by topics according to the following order: abstract, introduction, methodology, results, discussion, and conclusion.

Thank you for your attention to these revisions. We look forward to your feedback.

Sincerely,

The authors.

3. Have the authors made all data underlying the findings in their manuscript fully available?

The [PLOS Data policy](http://www.plosone.org/static/policies.action#sharing) requires authors to make all data underlying the findings described in their manuscript fully available without restriction, with rare exception (please refer to the Data Availability Statement in the manuscript PDF file). The data should be provided as part of the manuscript or its supporting information, or deposited to a public repository. For example, in addition to summary statistics, the data points behind means, medians and variance measures should be available. If there are restrictions on publicly sharing data—e.g. participant privacy or use of data from a third party—those must be specified.

A) **Abstract**

**Reviewer #2**

Overall: If the journal does not have special requirements, I recommend presenting the abstract section in order of background/objective, methods, results, conclusions.

Response:We would like to thank the reviewer for their valuable contributions, following the journal's guidelines (https://journals.plos.org/plosone/s/submission-guidelines), we made the changes to the abstract. We made an effort to follow the suggestions of reviewers 2 and 3 and maintain the number of words determined by the Journal. Thus, we begin the abstract from the objectives, in the methodology we detail the methodology used in the APC analyses In the results we present the total number of suicides evaluated during the study period, and we explain to readers what was considered statistically significant.

**Abstract**

**Objective**: To analyze the effect of age, period, and cohort on suicides among women by hanging, strangulation, suffocation, firearms, and autointoxication in different Brazilian regions from 1980 to 2019.**Methods:** Ecological time-trend study employing estimable functions to estimate APC models, facilitated through the Epi library of the R statistical program, version 4.2.1. Specific rates by age group per 100,00 women and relative risks by period and cohort were estimated using this method. **Results:** Between 1980 and 2019, 49,997 suicides among women were reported using the methods under study. Higher suicide rates per 100,000 women were observed in the South using strangulation and suffocation (2.42), while lower firearm suicide rates were observed in the Northeast (0.13). After adjusting the APC model, there was an increase in age-specific rates with advancing age across all regions for suicides by hanging, strangulation, and suffocation. In contrast, suicides by firearms and autointoxication showed a decrease in rates with advancing age. The period effect indicated an increased risk of suicides by hanging, strangulation (RR >1 and p<0.05) in the five-year intervals of the 2000s in the North, Southeast, and South regions. During the same period, there was an increased risk of suicides by autointoxication in the Southeast, South, and Northeast (RR>1, p<0.05). Suicides by firearms exhibited a statistically significant reduction in the risk of death from 2005 to 2019 in the Southeast and South regions, and from 2005 to 2014 in the Northeast and Midwest. The observed increase in the North region was not statistically significant (RR>1, p>0.05). The cohort effect demonstrated an increased risk of suicides by hanging, strangulation in younger cohorts (RR>1, p<0.05), whereas other methods showed an elevated risk in older cohorts relative to the 1950-1954 generation. **Conclusion:** The results presented here may suggest changes in suicide method preferences between 1980 and 2019.

**Key-words:** Suicide, women,Gender and Health; Mental Health; Age-period-cohort effect

- Line 25-27, Page 2: What is the meaning of this sentence placed here? Also, please add the research purpose.

Response: We restructured the abstract, removed this sentence and inserted the objective of the study.

**Objective**: To analyze the effect of age, period, and cohort on suicides among women by hanging, strangulation, suffocation, firearms, and self-poisoning in different Brazilian regions from 1980 to 2019.

- Line 31, Page 2: What is the purpose of using model estimation? Please provide a detailed description of the research design in the methodology section and what data indicators were collected. What is the sample size?

Response: We restructured the methodology as requested.

**Methods:** Ecological time-trend study employing estimable functions to estimate APC models, facilitated through the Epi library of the R statistical program, version 4.2.1. Specific rates by age group per 100,00 women and relative risks by period and cohort were estimated using this method.

- Line 31-45, Page 2: 1) Is this the results section? There are no transitional words. Please pay attention to the division of each section in the abstract to help readers clarify. 2) Please provide the numerical values of key indicators as evidence support, not just language expression. 3) Display the main results. Reduce excessive elaboration.

Response: We restructured the results as requested.

**Results:** Between 1980 and 2019, 49,997 suicides among women were reported using the methods under study. Higher suicide rates 100,000 women were observed in the South using strangulation and suffocation (2.42), while lower firearm suicide rates were observed in the Northeast (0.13). After adjusting the APC model, there was an increase in age-specific rates with advancing age across all regions for suicides by hanging, strangulation, and suffocation. In contrast, suicides by firearms and self-poisoning showed a decrease in rates with advancing age. The period effect indicated an increased risk of death (RR >1 and p<0.05) in the five-year intervals of the 2000s in the North, Southeast, and South regions. During the same period, there was an increased risk of suicides by self-poisoning in the Southeast, South, and Northeast (RR>1, p<0.05) and a reduction in the North (RR<1, p<0.05). Suicides by firearms exhibited a statistically significant reduction in the risk of death from 2005 to 2019 in the Southeast and South regions, and from 2005 to 2014 in the Northeast and Midwest. The observed increase in the North region was not statistically significant (RR>1, p>0.05). The cohort effect demonstrated an increased risk in younger cohorts (RR>1, p<0.05), whereas other methods showed an elevated risk in older cohorts relative to the 1950-1954 generation.

**Reviewer #3**

- I suggest the authors go direct to the goal of the paper.
- The results about age differences in suicide rates should be included.

Response: We thank you for your valuable contributions and have made the changes highlighted in red in the revised abstract, presented below.

**Abstract**

**Objective**: To analyze the effect of age, period, and cohort on suicides among women by hanging, strangulation, suffocation, firearms, and autointoxication in different Brazilian regions from 1980 to 2019.**Methods:** Ecological time-trend study employing estimable functions to estimate APC models, facilitated through the Epi library of the R statistical program, version 4.2.1. Specific rates by age group per 100,00 women and relative risks by period and cohort were estimated using this method. **Results:** Between 1980 and 2019, 49,997 suicides among women were reported using the methods under study. Higher suicide rates per 100,000 women were observed in the South using strangulation and suffocation (2.42), while lower firearm suicide rates were observed in the Northeast (0.13). After adjusting the APC model, there was an increase in age-specific rates with advancing age across all regions for suicides by hanging, strangulation, and suffocation. In contrast, suicides by firearms and autointoxication showed a decrease in rates with advancing age. The period effect indicated an increased risk of suicides by hanging, strangulation (RR >1 and p<0.05) in the five-year intervals of the 2000s in the North, Southeast, and South regions. During the same period, there was an increased risk of suicides by autointoxication in the Southeast, South, and Northeast (RR>1, p<0.05). Suicides by firearms exhibited a statistically significant reduction in the risk of death from 2005 to 2019 in the Southeast and South regions, and from 2005 to 2014 in the Northeast and Midwest. The observed increase in the North region was not statistically significant (RR>1, p>0.05). The cohort effect demonstrated an increased risk of suicides by hanging, strangulation in younger cohorts (RR>1, p<0.05), whereas other methods showed an elevated risk in older cohorts relative to the 1950-1954 generation. **Conclusion:** The results presented here may suggest changes in suicide method preferences between 1980 and 2019.

**Key-words:** Suicide, women,Gender and Health; Mental Health; Age-period-cohort effect

**B)Introduction**

**Reviewer #1**: The manuscript examines age, period, and cohort effects on suicide by methods used among women in Brazil.
I find the paper well-written; it has the potential to contribute to the existing literature on the topic. However, I have some concerns I would like the authors to consider.

- “These ideals are constantly conveyed to individuals through gender technologies performed by family, religion, school, art, media, among others [6,10,13,19]. Zanello [19] asserts that men's subjectivity is configured by gender technologies that emphasize the devices of sexual and economic efficiency, while women's subjectivity is shaped by love and maternal devices.”

  What do the authors consider as relevant gender technologies in this context? Please consider clarifying by using some examples.

Response: Thank you for the suggestion, we have inserted what was requested in the text, as shown in the text below.

The gender technologies are represented by linguistic codes and cultural representations such as cinema, media, games, and toys. These technologies are constantly performed by family, religion, school, art, media, and other institutions [6,10,13,19]. Zanello [19] asserts that men's subjectivity is configured by gender technologies that valorize the devices of sexual and economic efficacy, while women's subjectivity is shaped by the amorous and maternal devices. Thus, suicidal behaviors in men and women would be aligned with culturally defined gender scripts [20-23].

“…mortality in women according ccording to the most common means of perpetration…”

Please correct this sentence

Response: Correction made

Thus, the aim was to address the following research question: Are there differences in the effects of age, period, and cohort on suicide mortality in women according to the most common means of perpetration between and within Brazilian regions?

**Reviewer #2**

- Overall: Overall, the logic of the preface needs to be strengthened, and some keywords are not even elaborated in the preface section.

Response: We appreciate the reviewer's valuable suggestions, based on them and the suggestions of other reviewers, we made structural changes to this section of the manuscript. We discussed the issue of mental health in Brazil, not with regard to the National Suicide Prevention Policy, in addition, we expanded the definition of the effect of age, period and cohort.

- Previously, it was often mentioned that "the first two decades of the 1980s". Is there any difference or connection between the data from this period and the data from your research period?

Response: We believe the reviewer is referring to the one marked in red in the paragraph below. This period was included in our study, because we are evaluating the period from 1980 to 2019, the period from 2000 to 2019, including the first two decades of the 21st century. To make it clearer to readers, we decided to make a change in the text.

- Line 52, page 3: Complex? Where is it reflected? The first paragraph only shows the high incidence of suicidal behavior. Please modify and improve. Also, this is a new paragraph, what does "it" refer to? This statement does not seem to have a causal relationship with the preceding text.

Line 53-54, Page 3: What is the significance of emphasizing the different impacts on different genders? Where are the different impacts reflected? This is abrupt. Please modify.

- Line 58-60, Page 3: Please use appropriate transitional sentences to continue the two paragraphs.

Response Based on the suggestions above, we rewrote the first three paragraphs of the introduction.

**In the text**

Suicide affects people from different backgrounds, ethnical groups, socio-economic status and geographical locations [1–6]. Suicide can be defined as any deliberated act by which an individual’s death results directly or indirectly from a self-inflicted injury or poisoning [1-6]. Considered a sociocultural phenomenon, it is attributed to the interaction of individual and contextual factors [1-6]. At the individual level, noteworthy factors include depression, bipolar affective disorder, schizophrenia, anxiety, prior history of suicide attempts, excessive alcohol and drug use, and philosophical and existential questions [7-11]. Contextual factors include economic and health crises, as well as societies characterized by fragility in social cohesion [7-11].

The fragility in social cohesion has been documented in patriarchal societies with high levels of inequality in gender, race, and class relations [7,12-16]. In these societies, the illness and death of women and men are influenced by inequalities in gender relations [14-16]. The construct of gender is an inherent dimension of societal life, subject to various definitions over time [15-19]. Joan Scott [17] argues that gender emerges from the relationship of submission and oppression of women by men, shaping the notions of being male and being female in these societies. In other words, it defines what the thoughts, feelings, and behaviors of women and men should be through the ideal notions of masculinity and femininity, which are presented to individuals via gender technologies [6,10,13,19].

The gender technologies are represented by linguistic codes and cultural representations such as cinema, media, games, and toys. These technologies are constantly performed by family, religion, school, art, media, and other institutions [6,10,13,19]. Zanello [19] asserts that men's subjectivity is configured by gender technologies that valorize the devices of sexual and economic efficacy, while women's subjectivity is shaped by the amorous and maternal devices. Thus, suicidal behaviors in men and women would be aligned with culturally defined gender scripts [20-23].

- Line 86-87, Page 4: Please elaborate more on "crucial". And clarify the significance of conducting this research.

Response: Based on the reviewers' suggestions, we made a change in the structure of the text, to make clear the importance of ecological studies of temporal trends in the evaluation and planning of suicide prevention policies.

**In the text**

Understanding the most commonly used methods of suicide is an important tool for the development of strategies to prevent future deaths. The WHO document (LIVE LIFE: An Implementation Guide for Suicide Prevention in Countries) asserts that effective suicide prevention measures necessarily involve restricting access to means of suicide [34]. This is only feasible when the behavior of the population in choosing methods over time is well understood. The temporal evolution of suicides among women may exhibit significant differences according to age group, study period, generation, and means of suicide [31-33]. Therefore, in studies on the temporal trends of suicides by method, it is important to analyze the effects of three temporal factors (age, period, and cohort) on the behavior of the time series [30-33, 35].

- Line 92-97, Page 5: The introduction of the keywords "age period cohort effect" or "age period cohort" in this paragraph is too concise. Please provide more explanation to assist in explaining the significance of the research.

Response: Based on the reviewers' suggestions, we made a change in the structure of the text, to make clear the importance of ecological studies of temporal trends in the evaluation and planning of suicide prevention policies. And we've included more details about what the age, period, and cohort effects are.

**In the text**

The age effect represents changes in the risk of illness and death associated with different age groups, which may arise due to physiological changes, accumulation of social experience, social roles, changes in social status, or a combination of these factors. The period effect involves structural transformations that affect all age groups simultaneously, such as major sociocultural, economic, and environmental changes (major wars, pandemics, economic crises, health policies, policies restricting access to means of suicide, and policies expanding access to mental health services, among others). The cohort effect manifests as members of different cohorts experience the impact of these sociocultural, economic, and environmental changes differently throughout their lives. Therefore, exposure to risk and protective factors in the development of diseases and health conditions varies according to age group [33,35-36].

- Line 102, Page 5: Please propose reasonable research hypotheses based on your research objectives.

Response: We inserted the objective of the study at the end of the introduction.

**In the text**

The age effect represents changes in the risk of illness and death associated with different age groups, which may arise due to physiological changes, accumulation of social experience, social roles, changes in social status, or a combination of these factors. The period effect involves structural transformations that affect all age groups simultaneously, such as major sociocultural, economic, and environmental changes (major wars, pandemics, economic crises, health policies, policies restricting access to means of suicide, and policies expanding access to mental health services, among others). The cohort effect manifests as members of different cohorts experience the impact of these sociocultural, economic, and environmental changes differently throughout their lives. Therefore, exposure to risk and protective factors in the development of diseases and health conditions varies according to age group [33,35-36].

Considering the importance of the age, period, and cohort effects on the temporal evolution of suicide method choices[33-34], the objective of this study is to analyze the effects of age, period, and cohort on suicides according to the most commonly used methods in Brazil, specifically hanging, strangulation/suffocation (HSS), firearms (FA), and autointoxication (AUT) across Brazilian regions from 1980 to 2019.

- As you mentioned in your keyword about mental health, it was not elaborated on in the introduction section.

Response: We introduced the discussion on mental health in the paragraph that discussed the interaction between sociodemographic factors, gender-based violence, gender norms, inequities and lack of access policies to mental health and suicidal behavior.
**In the text**

In this context, gender-based violence emerges as a significant factor related to suicidal behavior in women. Devries et al. (2011) argue that suicidal behavior in women can be explained by the interaction between sociodemographic factors (including age, marital status, migration, and socioeconomic status), inequities or lack of access to mental health services, history of childhood violence, experiences of violence in adulthood, and the presence of gender norms in society (such as polygamy, dowry, and family-arranged marriages, among other practices) [12].

Brazil is a country with high gender inequality; women earn only 76% of the average income of men, despite having higher levels of education. They also have limited representation in politics and in managerial positions in public and private enterprises [24]. Furthermore, the country is considered one of the most violent for women, maintaining high rates of physical, sexual, and psychological violence, as well as femicides and female homicides [24-25].

Violence against women in Brazil is a historical problem that persists despite the implementation of legislation in the 2000s aimed at punishing offenders and protecting women in situations of violence. This includes stricter laws on femicide and the National Policy for the Reduction of Morbidity and Mortality from Accidents and Violence, which guided the health sector’s response to these health concerns [26-28]. However, the implementation of these laws has not been matched by an increase in the budget for building a network of protection and support services for women in situations of violence [24,28]. Consequently, more than a decade after the implementation of these policies, Brazil still has an insufficient number of specialized services to assist women in situations of sexual violence, shelter homes, specialized mental health care services for victims, and specialized police stations for women in situations of violence [24].

**Reviewer #3**

Dear authors,
I really enjoy reading your paper. Addressing changes in suicide methods adopted by women over the time and the regional differences are really important to implement public policies to fight this public health problem. However, bellow I include some some suggestions to improve this manuscript.

- I suggest the authors excluding lines 92-102 since the subjects were addressed in paragraphs above.

Response: We restructured the introduction according to the reviewers' suggestions and removed the paragraphs that contained the same information.

**In the text**

Understanding the most commonly used methods of suicide is an important tool for the development of strategies to prevent future deaths. The WHO document (LIVE LIFE: An Implementation Guide for Suicide Prevention in Countries) asserts that effective suicide prevention measures necessarily involve restricting access to means of suicide [34]. This is only feasible when the behavior of the population in choosing methods over time is well understood. The temporal evolution of suicides among women may exhibit significant differences according to age group, study period, generation, and means of suicide [31-33]. Therefore, in studies on the temporal trends of suicides by method, it is important to analyze the effects of three temporal factors (age, period, and cohort) on the behavior of the time series [30-33, 35].

The age effect represents changes in the risk of illness and death associated with different age groups, which may arise due to physiological changes, accumulation of social experience, social roles, changes in social status, or a combination of these factors. The period effect involves structural transformations that affect all age groups simultaneously, such as major sociocultural, economic, and environmental changes (major wars, pandemics, economic crises, health policies, policies restricting access to means of suicide, and policies expanding access to mental health services, among others). The cohort effect manifests as members of different cohorts experience the impact of these sociocultural, economic, and environmental changes differently throughout their lives. Therefore, exposure to risk and protective factors in the development of diseases and health conditions varies according to age group [33,35-36].

- I didn’t see nothing about policies in fighting gender inequities or promoting women health in Brazil in the introduction and in discussion. It is essential to this paper to address this theme

Response: We enter the discussion about policies in fighting gender inequities or promoting women health in Brazil in the introduction and in discussion.

**In the text**

In this context, gender-based violence emerges as a significant factor related to suicidal behavior in women. Devries et al. (2011) argue that suicidal behavior in women can be explained by the interaction between sociodemographic factors (including age, marital status, migration, and socioeconomic status), inequities or lack of access to mental health services, history of childhood violence, experiences of violence in adulthood, and the presence of gender norms in society (such as polygamy, dowry, and family-arranged marriages, among other practices) [12].

Brazil is a country with high gender inequality; women earn only 76% of the average income of men, despite having higher levels of education. They also have limited representation in politics and in managerial positions in public and private enterprises [24]. Furthermore, the country is considered one of the most violent for women, maintaining high rates of physical, sexual, and psychological violence, as well as femicides and female homicides [24-25].

Violence against women in Brazil is a historical problem that persists despite the implementation of legislation in the 2000s aimed at punishing offenders and protecting women in situations of violence. This includes stricter laws on femicide and the National Policy for the Reduction of Morbidity and Mortality from Accidents and Violence, which guided the health sector’s response to these health concerns [26-28]. However, the implementation of these laws has not been matched by an increase in the budget for building a network of protection and support services for women in situations of violence [24,28]. Consequently, more than a decade after the implementation of these policies, Brazil still has an insufficient number of specialized services to assist women in situations of sexual violence, shelter homes, specialized mental health care services for victims, and specialized police stations for women in situations of violence [24].

**C)Materials and methods**

**Reviewer #1**

- The authors have included in their study women aged ten years and older. While it is quite a frequent practice to combine data on adults and adolescents when examining suicide, there is ample evidence showing significant differences in the suicide behavior of adolescents compared to adults. Why did the authors decide to include the population aged 10–19? Taking into account the potentially different etiology behind suicide in adolescents, please consider excluding these age groups or justifying in the text the decision to include them. In addition, among the limitations, the authors mentioned fewer observations in younger generations.

**Response**

When analyzing the suicide of women in Brazilian regions (APC) according to the means of perpetration, we are prompted to do so from a gender relations perspective. It should be noted that suicide among young people represents an even greater taboo in Brazilian society when compared to suicide among adults (Galvão and Silva, 2023; Brazil, 2023; Dantas et al., 2021; Dantas et al., 2023). The inclusion of the 10-19 age range is justified by the following reasons: 1) Women in this age group already commit suicide (Galvão and Silva, 2023; Brazil, 2023; Dantas et al., 2021; Dantas et al., 2023); 2) It is necessary to analyze all age groups, as by doing so, we will recognize the differences in the use of suicide methods among different generations of women (Galvão and Silva, 2023; Brazil, 2021; Dantas et al., 2021; Dantas et al., 2023); 3) We know and acknowledge that children, adolescents, adults, and the elderly demonstrate sociocultural differences in suicidal behavior. However, our intention is to show a global panorama without excluding these differences, which are also related to the concept of gender that marks our study; 4) In Brazil, children and adolescents in this age range are already exposed to complex situations (sexual violence, child marriage, etc.) that may influence their deaths by suicide (Galvão and Silva, 2023; Brazil, 2023; Dantas et al., 2021; Dantas et al., 2023; Goes et al., 2023). Lastly, we follow other studies conducted in Brazil on this topic that include this age range (our studies and others), and we are aligned with the Ministry of Health, which in its epidemiological bulletins on mortality by suicide among men and women, uses all the age groups of the present study.

In all studies involving the APC method to assess the number of observations in cohorts, regardless of the object of study, we will observe a smaller number of observations in the initial and final cohorts (as shown in the table below). However, this characteristic is not a limitation for including them in studies. The only recommendation given by researchers of this method is to avoid using these cohorts as the reference cohort. Instead, one should choose median cohorts for reference as they present a higher number of observations (Holford, 1983; Holford, 2001; Yang and Land, 2013).

Table. Example of cohort data structure by age group.

**Reference**

1. Baére F, Zanello V. O gênero no comportamento suicida: uma leitura epidemiológica dos dados do Distrito Federal. Estud Psicol 2018; 23(2):168-17
2. Saffioti HIB. Gênero, patriarcado, violência. 2ª ed. São Paulo: Editora Perseu Abramo; 2004.
3. Segato RL. Gênero e colonialidade: em busca de chaves de leitura e de um vocabulário estratégico descolonial. E-Cadernos Ces 2012; 18:1-5.
4. Dantas ESO, Farias YMF, Resende EB, Santos GWS, Silva PG, Meira KC. Estimates of suicide mortality in women residents in northeast brazilian states from 1996 to 2018. Ciencia & saude coletiva. 2021; 26:4795–4804. https://doi.org/10.1590/1413-812320212610.29552020 PMID: 34730664
5. Galvão PVM, da Silva CMFP. Analysis of age, period, and birth cohort effects on suicide mortality in Brazil and the five major geographic regions. BMC Public Health. 2023 Jul 13;23(1):1351. doi: 10.1186/s12889-023-16289-0. PMID: 37442995; PMCID: PMC10347758.
6. Dantas ESO, Meira KC, Bredemeier J, Amorim KPC. Suicide among women in Brazil: a necessary discussion from a gender perspective. Cien Saude Colet. 2023 May;28(5):1469-1477. Portuguese, English. doi: 10.1590/1413-81232023285.16212022. Epub 2022 Oct 22. PMID: 37194879.
7. Brasil, Ministério da Saúde, Secretaria de Vigilância em Saúde. Mortalidade por suicídio e notificações de lesões autoprovocadas no Brasil. Boletim Epidemiológico, Brasília [Internet]. 2021 [cited jun 2023].
8. Goes EF, Ferreira AJF,Meira KC, Myrrha LJD, Reis AP, Nunes VGA et al.Desigualdades raciais nas tendências da maternidade adolescente e no acesso ao pré-natal no Brasil, 2008-2019. Research, Society and Development 2023; 12 (1): e8312139404
9. Holford TR. Approaches to fitting age-period-cohort models for the Lexis diagram. Stat Med. 2006; 26:3018–45. 15.
10. Yang Y, Land KC. Age-Period-Cohort Analysis: New models, methods, and empirical applications. CRC Press: North Carolina, 2013

Given this, we decided to maintain the age groups of 10-14 years and 15-19 years in our study. In the methodology, we justify the choice of these age groups. In the discussion, we delve deeper into the factors related to suicide in different cohorts of women. We also clarify the reason for the limitation regarding the small number of observations in the younger cohorts and why we decided to include them in the analyses.

- Table 1: “W75 e W76”

  Please correct the typographical error.

Response

We performed the indicated correction and changed the table formatting as shown below.

**Table 1.** Death records sourced from the Mortality Information System of the SUS Information Technology Department, categorized by ICD-9 and ICD-10.

| **Health problem** | **ICD-9** | **ICD-10** |
| --- | --- | --- |
| **Suicide** |  |  |
| Suicide by firearm | E955 | X72 to X74 |
| **Other external causes of accidental injuries** |  |  |
| Other external causes of accidental injuries by hanging, strangulation, and suffocation. | E913 | W75 toW76 |
| Other external causes of accidental injuries by accidental autointoxication. | E850 to E869 | X40 to X49 |
| Other external causes of accidental injuries by firearm | E922 | W32 to W34 |
| **Assault** |  |  |
| Homicide by hanging/strangulation/suffocation | E963 | X91 |
| Homicide by autointoxication | E962 | X85 to X90 |
| Homicide by firearm | E965 | X93 to X95 |
| **Event of undetermined intent** |  |  |
| Hanging/strangulation/suffocation, intent undetermined | E983 | Y20 |
| Intoxication or autointoxication, intent unknown (whether intentional or accidental) | E980 to E982 | Y1 to Y19 |
| Firearm discharge, rifle, pistol, other firearms, intent undetermined | E985 | Y22 to Y24 |
| Sequelae of self-inflicted injury | E959 | Y87 |

Source: 9th and 10th editions of the International Classification of Diseases and Related Health Problems.

- “Here, i = 1, ..., I; j = 1, ... J; k = 1, ..., K; and where K = I + J-1.”

  Since lowercase letters i, j, and k are only used in the equation presented later in the text, this sentence might make more sense after introducing Holford's proposal.

  6.) “… the effect of period …”

  Unlike in the main text, this is in the equation.

Where $E[r_{ij}]$ denotes the expected rate, $\theta_{ij}$ the number of observed deaths, and $N_{ij}$ the population at risk of death in age group $i$ and period $j$. The parameter $\mu$ represents the average effect, $\alpha_{i}$ represents the effect of age group $i$, $\beta_{ij}$ the effect of period $j$, and $\gamma_{k}$the effect of cohort $k (k = 1,..., k = I + J - 1 = 22)$ and$\varepsilon_{ij}$ represents the random error in age (i) and period (j).

- “cohort (k = 1, . . . , K= I + J − 1 = 22)”

  Here, the second lowercase k in the brackets seems to be a typographical error.

Response: We appreciate the suggestions and make the requested changes.

**In the text**

The APC effects were estimated using regression models with a Poisson distribution for the number of observed deaths in each age group i and period j (θij). These effects are additively related to the logarithm of the expected mortality rate (E(rij)), following Holford's proposal [35].

$$ln\left( E\left[ r_{ij} \right] \right)=ln\left( \frac{\theta_{ij}}{N_{ij}} \right)=\mu+\alpha_{i}+\beta_{j}+\gamma_{k}+\varepsilon_{ij}$$

Where $E[r_{ij}]$ denotes the expected rate, $\theta_{ij}$ the number of observed deaths, and $N_{ij}$ the population at risk of death in age group $i$ and period $j$. The parameter $\mu$ represents the average effect, $\alpha_{i}$ represents the effect of age group $i$, $\beta_{ij}$ the effect of period $j$, and $\gamma_{k}$the effect of cohort $k (k = 1,...K; K = I + J - 1 = 22)$ and$\varepsilon_{ij}$ represents the random error in age (i) period (j), and cohort ($k)$. Here, i = 1, ..., I; j = 1, ... J; k = 1, ..., K. Where I correspond to the number of age groups, J to the number of periods, and K to the number of cohorts. Consequently, in this study we obtained I = 15 age groups, J = 8 periods, and K = I + J – 1 = 22 birth cohorts (1900 to 2009).

Reviewer #2: ID: PONE-D-24-08405

- Study design and data sources
  Line 107, Page 5: Please provide a detailed explanation of which recommendations.the *Guidelines for Accurate and Transparent Health Estimates Reporting*: the GATHER statement [32].

Response: We chose to include supplementary material in which we inserted how we responded to the guideline request in the manuscript.

**Int the text**

**Study design**

An observational study with an ecological time-trend design was conducted to analyze the effect of age, period, and cohort on suicides among women by hanging, strangulation/suffocation, self-poisoning, and firearm from 1980 to 2019 in Brazilian regions. This study followed the recommendations of the Guidelines for Accurate and Transparent Health Estimates Reporting (GATHER) statement, describing the data sources (death records and population), selected variables, calculated indicators, method of analysis, and the description and discussion of the results [32], as detailed in S1 Table.

- The research design is too brief to fully understand your research ideas and strive for perfection.

Response : We rewrote the description of the study design to meet the reviewer's request.

**Int the text**

**Study design**

An observational study with an ecological time-trend design was conducted to analyze the effect of age, period, and cohort on suicides among women by hanging, strangulation/suffocation, self-poisoning, and firearm from 1980 to 2019 in Brazilian regions. This study followed the recommendations of the Guidelines for Accurate and Transparent Health Estimates Reporting (GATHER) statement, describing the data sources (death records and population), selected variables, calculated indicators, method of analysis, and the description and discussion of the results [32], as detailed in S1 Table.

- Study variables:
  I don't quite understand. The title is called Study Variables, but the content does not clearly summarize all the research variables and introduce the collection of key variables. Please indicate which variables and indicators were collected for this study.

Response: We rewrote the methodology following GATHER recommendations.

**Int the text**

**Data sources and Study variables**

The data used in this study were freely accessed from the Mortality Information System of the SUS Department of Informatics (SIM/DATASUS) [34]. This system is universally accessible. SIM/DATASUS is the information system of the Brazilian Ministry of Health, which provides death records for all Brazilian states and municipalities since 1979. In the present study, the microdata for each Brazilian region was collected annually from 1980 to 2019.

The microdata is available in dbc format and was converted to dbf format using the Tabwin program version 4.15 for Windows provided by the Brazilian Ministry of Health. After converting the data to dbf format, the death records for each year (1980 to 2019) were aggregated for each of the Brazilian regions using the R software (version 4.1), extracting only death records of females aged 10 years and older. Death records were extracted from the Mortality Information System of the Department of Informatics of the Unified Health System (SIM/DATASUS) [34], considering the 9th and 10th editions of the International Classification of Diseases and Related Health Problems (ICD-9 and ICD-10), as per the codifications presented in Table 1.

Population data for mortality rate estimates were also obtained from DATASUS in the sociodemographic and economic data section, based on a demographic census from 1980, 1991, 2000 and 2010. The Brazilian Institute of Geography and Statistics estimated populations projections on July 10 of the intercensal years [38].

We analyzed all death records classified as suicides in women aged 10 and older, utilizing the methods of hanging/strangulation/suffocation, firearm, or autointoxication. These incidents occurred from 1980 to 2019 across the five major regions of the country.

The variables collected pertained to geographic region (North, Northeast, Southeast, South, and Midwest), age group (10 to 14, 15 to 19, 20 to 24, 25 to 29, 30 to 34, 35 to 39, 40 to 44, 45 to 49, 50 to 54, 55 to 59, 60 to 64, 65 to 69, 70 to 74, 75 to 79, and 80 years or older), year of death (1980 to 2019), and health problem (Suicide, Other external causes of accidental injuries, Assault, and Event of undetermined intent).

Age groups from 10 to 14 years to 80 years or older were chosen due to an excess of zeros in smaller age groups. We opted to include the age group of 10 to 19 years because, in Brazil, an increase in suicide rates has been observed in this age group over the last decade [29,31-33]. Additionally, Brazilian children and adolescents, particularly in regions with greater socioeconomic vulnerability, in this age range are already exposed to complex situations such as sexual violence, child marriage, and teenage pregnancy, which may influence their deaths by suicide [15,19,39,40].

**Correction for poor certification and underreporting of death records**

The Mortality Information System (SIM) exhibits varying quality in death certification, coverage, and completeness of variables among Brazilian regions. Notably, there is a high proportion of death records with an undetermined underlying cause and significant underreporting, particularly in the North and Northeast regions [41-44]. Faced with this, we chose to rectify death records for both poor certification and underreporting. Since there is no consensus in the literature regarding the best method to correct for the poor certification of suicides [30-31,33], we adapted the approach proposed by Garcia et al. [45] in six steps, as outlined in S1Table. The results of the stages of the death record correction process are presented in S2 Table.

After correcting for poorly certified deaths, we addressed underreporting by utilizing the degree of coverage of death records for women, segmented by geographic region and decade. This coverage was estimated using the Adjusted Synthetic Extinct Generations (SEG-adj) death distribution method, which combines the extinct generations and general balancing equation methods [44,46]. The estimated coverage degree was provided by researchers from the Population Estimates and Projections Laboratory at the Federal University of Rio Grande do Norte [47]. The rectification process of the records was conducted by two independent researchers and verified by a third.

**Statistical analyses**

**Exploratory analyses**

Following the correction of death records, we calculated specific mortality rates by age group, both crude and standardized using the direct method, with the World Health Organization's proposed global standard population. Mortality coefficients per 100,000 women were computed based on the means of perpetration (firearm, autointoxication, and hanging/strangulation/suffocation). To assess the effect of age, period, and cohort, we grouped age ranges and periods into five-year intervals: age group (10 to 14, 15 to 19, 20 to 24, 25 to 29, 30 to 34, 35 to 39, 40 to 44, 45 to 49, 50 to 54, 55 to 59, 60 to 64, 65 to 69, 70 to 74, 75 to 79, and 80 years or older,) period (1980 to 1984,1985 to 1989,1990 to 1994, 1995 to 1999, 2000 to 2004, 2005 to 2009, 2010 to 2014 and 2015 to 2019), and cohort (1900 to 1904 , 1905 to 1909, 1910 to 1914, 1910 to 1914, 1915 to 1919, 1920 to 1924, 1925 to 1929, 1930 to 1934, 1935 to 1934, 1940 to 1944, 1945 to 1949, 1950 to 1954, 1955 to 1959, 1960 to 1964, 1965 to 1969, 1970 to 1974, 1975 to1979, 1980 to 1984, 1985 to 1989, 1990 to 1994, 1995 to 1999, 2000 to 2004 and 2005 to 2009).

In the descriptive analysis of the temporal evolution of mortality rates by year and method, we smoothed the corrected suicide mortality coefficients using triennial moving averages, categorized by means of perpetration (hanging/strangulation/suffocation, firearm, and autointoxication). After smoothing the rates, line graphs were constructed to assess the temporal evolution of mortality rates [42].

**Age, period and cohort model**

The APC effects were estimated using regression models with a Poisson distribution for the number of observed deaths in each age group i and period j (θij). These effects are additively related to the logarithm of the expected mortality rate (E(rij)), following Holford's proposal [35].

$$ln\left( E\left[ r_{ij} \right] \right)=ln\left( \frac{\theta_{ij}}{N_{ij}} \right)=\mu+\alpha_{i}+\beta_{j}+\gamma_{k}+\varepsilon_{ij}$$

Where $E[r_{ij}]$ denotes the expected rate, $\theta_{ij}$ the number of observed deaths, and $N_{ij}$ the population at risk of death in age group $i$ and period $j$. The parameter $\mu$ represents the average effect, $\alpha_{i}$ represents the effect of age group $i$, $\beta_{ij}$ the effect of period $j$, and $\gamma_{k}$the effect of cohort $k (k = 1,...K; K = I + J - 1 = 22)$ and$\varepsilon_{ij}$ represents the random error in age (i) period (j), and cohort ($k)$. Here, i = 1, ..., I; j = 1, ... J; k = 1, ..., K. Where I correspond to the number of age groups, J to the number of periods, and K to the number of cohorts. Consequently, in this study we obtained I = 15 age groups, J = 8 periods, and K = I + J – 1 = 22 birth cohorts (1900 to 2009).

The estimation of APC effects has a primary limitation known as the non-identifiability problem of parameters in the complete model due to the linear relationship among temporal effects (I= J - K). Various methodological tools have been proposed to address this limitation, but there is no consensus in the literature on the best methodology. In this study, APC effects were estimated using estimable functions, as proposed by Holford [35-36] and implemented in models adjusted by the Epi library (<https://CRAN.R-project.org/package=Epi>) in the R software (<https://www.R-project.org/>) [49].

Estimable functions are limited to the analysis of linear combinations and curvature effects of temporal terms (age, period, and cohort). The linear trend of effects is divided into two components: the linear effect of age and the drift effect (linear effect of period and cohort). The longitudinal trend of age is the sum of age and period slope (αL + βL), where αL and βL are the linear trends of age and period, respectively. The second drift term represents the linear trend of the logarithm of specific rates (mortality) by age and is equal to the sum of period and cohort slopes (βL + γL), where βL and γL are the linear trends of period and cohort, respectively [35,49].

Fifteen APC analyses (scenarios) were conducted for female suicides by firearm, autointoxication, and hanging/strangulation/suffocation in the five Brazilian regions. In each scenario, the adjusted APC submodels were compared in a nested manner via deviance statistics and likelihood ratio tests at a 5% significance level, as proposed by Holford [35,49].

The deviance analysis of the APC models determined by the Epi library estimates six nested equations, namely: (1) f(a) age; (2) f(a) + δc age-drift; (3) f(a) + h(c) age-cohort; (4) f(a) + g(p) + h(c) age-period-cohort; (5) f(a) + g(p) age-period; and (6) f(a) + δp age-drift. Here, a, p, and c represent the effects of age, period, and cohort; f, h, and g are smooth functions of parameters; and δ is a linear effect [35,49].

Based on the best-fit model, estimated specific mortality rates by age and relative risks (RR) were extracted for each period and cohort concerning their respective reference categories (period: 2000-2004; birth cohort: 1950-1954). Interval estimates were obtained at a 95% confidence level [35,49]. We chose the five-year period 2000-2004 as it marks the implementation of significant suicide prevention measures (National Mental Health Policy and Disarmament Statute and National Policy for the Reduction of Morbidity and Mortality from Accidents and Violence) [27,33,50-51]. Regarding the reference cohort, we selected 1950-1954 because median cohorts tend to have a greater quantity of values, being more stable and complete than the first and last ones [35-36,49]. Furthermore, previous studies conducted in Brazil have shown a lower risk of death in generations born from the 1950s onwards [33,50-51].

- Table 1: A three-line table is recommended if not specifically required by the journal.

**Table 1.** Death records sourced from the Mortality Information System of the SUS Information Technology Department, categorized by ICD-9 and ICD-10.

| **Health problem** | **ICD-9** | **ICD-10** |
| --- | --- | --- |
| **Suicide** |  |  |
| Suicide by firearm | E955 | X72 to X74 |
| **Other external causes of accidental injuries** |  |  |
| Other external causes of accidental injuries by hanging, strangulation, and suffocation. | E913 | W75 toW76 |
| Other external causes of accidental injuries by accidental autointoxication. | E850 to E869 | X40 to X49 |
| Other external causes of accidental injuries by firearm | E922 | W32 to W34 |
| **Assault** |  |  |
| Homicide by hanging/strangulation/suffocation | E963 | X91 |
| Homicide by autointoxication | E962 | X85 to X90 |
| Homicide by firearm | E965 | X93 to X95 |
| **Event of undetermined intent** |  |  |
| Hanging/strangulation/suffocation, intent undetermined | E983 | Y20 |
| Intoxication or autointoxication, intent unknown (whether intentional or accidental) | E980 to E982 | Y1 to Y19 |
| Firearm discharge, rifle, pistol, other firearms, intent undetermined | E985 | Y22 to Y24 |
| Sequelae of self-inflicted injury | E959 | Y87 |

Source: 9th and 10th editions of the International Classification of Diseases and Related Health Problems.

Reviewer#3:
Methods
I only suggest you to include your data sheet in a repository where others can have free access.

**Response**

The databases and R codes will be made available on the Zenodo platform.

**D) Results**

Reviewer #1

- All results were shown after correcting the death records. Did the authors assess the trends – not the death rates – using the data before correction? Were there any notable differences between trends based on corrected versus uncorrected records (including potential regional differences)?

**Response**

We conducted an Age-Period-Cohort (APC) effect analysis using both corrected and uncorrected data. The trend over the four decades of study, analyzed through the age-drift trend, showed differences between corrected and uncorrected rates in firearm suicides in the South and self-poisoning suicides in the Southeast. In the South region, uncorrected rates for firearm suicides showed an upward trend (RR=1.04, p<0.05) while corrected rates showed a downward trend (RR=0.96,p<0.05). In the Southeast, uncorrected rates for self-poisoning suicides showed an upward trend (RR=1.01,p<0.05), while corrected rates showed a downward trend (RR=0.99, p<0.05).

We believe these findings may be related to the impact of improved data quality starting in the 2000s, which may have led to an increase in rates compared to earlier periods when data quality was poorer. However, when analyzing the period effect between corrected and uncorrected rates, we observed changes in the risk profile for mortality in the period effect, as shown in the following tables. These differences may be related to the poor quality of death records in the Brazilian Mortality Information System. In this system, there is an observed relationship between the temporal trend of defined external causes (accidental, self-inflicted, and intentional external causes) and records classified as events of indeterminate intent. When the latter increases, the former decreases (Brasil, 2014; Brasil, 2021; Souza et al., 2014; Cerqueira, 2020; Cerqueira, 2022).To provide more accurate estimates, we chose to perform our analyses using the corrected mortality records because the Brazilian Mortality Information System presents limitations in the quality and coverage of death records, especially in locations with high socioeconomic vulnerability (Szwarcwald et al., 2011; Brasil, 2014; Mello; Jorge, 2002; Queiroz et al., 2017). Thus, to obtain more reliable estimates of mortality according to causes, the application of indirect correction techniques is recommended (Szwarcwald et al., 2011; Brasil, 2014; Mello; Jorge, 2002; Queiroz et al., 2017; Dantas et al., 2021; Santos et al., 2014). Considering the lack of consensus in the literature regarding the best correction method for death records classified as suicide (Dantas et al., 2021; Rockett et al., 2010; Santos et al., 2014), in this study, we adapted the correction method proposed by Garcia et al. (2015).

Table 1. Hanging, strangulation and suffocation

The differences in period effects between uncorrected and corrected data are highlighted in red.

| **Hanging, strangulation and suffocation** | | |
| --- | --- | --- |
| **Period** | **North (incorrect)** | **North (correct)** |
| 1980 to 1984 | 1.12 ( 0.96- 1.31) | **1.20( 1.05-1.36)*** |
| 1985 to 1989 | 1.08 0.97 -1.20) | **1.13( 1.04- 1.23)*** |
| 1990 to 1994 | 1.05 ( 0.99- 1.11) | **1.07( 1.02- 1.12)*** |
| 1995 to 1999 | 1.01( 0.99- 1.03) | 1.02( 1.00- 1.03) |
| 2000 to 2004 | **Reference** | **Reference** |
| 2005 to 2009 | 1.017 ( 0.96-1.07) | 1.04 (0.99- 1.09) |
| 2010 to 2014 | 1.07( 0.92- 1.24) | **1.14( 1.01- 1.29)*** |
| 2015 to 2019 | 1.15( 0.99- 1.34) | **1.21 ( 1.07-1.37)*** |
| **Period** | **Northeast (incorrect)** | **Northeast (correct)** |
| 1980 to 1984 | **0.90( 0.82- 0.99)*** | 1.04( 0.96- 1.13) |
| 1985 to 1989 | **0.93( 0.87- 0.99)*** | 1.003( 0.95- 1.05) |
| 1990 to 1994 | **0.96 ( 0.93- 0.99)*** | **0.96( 0.93- 0.98)*** |
| 1995 to 1999 | 0.98( 0.97- 1.00) | **0.93( 0.91- 0.96)*** |
| 2000 to 2004 | **Reference** | **Reference** |
| 2005 to 2009 | 0.98-( 0.93 -1.03) | **1.13( 1.06- 1.19)*** |
| 2010 to 2014 | 0.94( 0.86- 1.02) | 0.99( 0.92- 1.06) |
| 2015 to 2019 | **0.88( 0.813-0.96)*** | 0.97(0.90- 1.04) |
| **Period** | **Southeast (incorrect)** | **Southeast (correct)** |
| 1980 to 1984 | **1.26( 1.17- 1.35)*** | **1.21( 1.13- 1.29)*** |
| 1985 to 1989 | **1.17( 1.12- 1.22)*** | **1.16( 1.11- 1.20)*** |
| 1990 to 1994 | **1.09 ( 1.06- 1.13)*** | **1.11( 1.07- 1.14)*** |
| 1995 to 1999 | 1.03( 1.00- 1.06) | **1.06( 1.02- 1.09)*** |
| 2000 to 2004 | **Reference** | **Reference** |
| 2005 to 2009 | 1.00( 0.96- 1.06) | 0.96( 0.92- 1.00) |
| 2010 to 2014 | **1.12( 1.04- 1.19)*** | **1.09( 1.02- 1.16)*** |
| 2015 to 2019 | **1.34 (1.27-1.43)*** | **1.35( 1.28- 1.44)*** |
| **Period** | **South (incorrect)** | **South (correct)** |
| 1980 to 1984 | **1.17( 1.10- 1.25)*** | **1.11( 1.04- 1.18)*** |
| 1985 to 1989 | **1.21( 1.15- 1.27)*** | **1.14( 1.08- 1.21)*** |
| 1990 to 1994 | **1.23( 1.16- 1.29)*** | **1.17( 1.09- 1.25)*** |
| 1995 to 1999 | **1.16 ( 1.11- 1.21)*** | **1.11( 1.06- 1.17)*** |
| 2000 to 2004 | **Reference** | **Reference** |
| 2005 to 2009 | 0.98( 0.95- 1.01) | 1.01 ( 0.96- 1.04) |
| 2010 to 2014 | **1.23 (1.15- 1.33)*** | **1.11( 1.03- 1.19)*** |
| 2015 to 2019 | **1.30 ( 1.22- 1.39)*** | **1.23 ( 1.15- 1.31)*** |
| **Period** | **Midwest (incorrect)** | **Midwest (correct)** |
| 1980 to 1984 | **0.59( 0.51- 0.68)*** | **0.66 ( 0.57- 0.76)*** |
| 1985 to 1989 | **0.69( 0.62- 0.76)*** | **0.76 ( 0.70- 0.84)*** |
| 1990 to 1994 | **0.80( 0.75-0.85)*** | **0.88 ( 0.84- 0.92)*** |
| 1995 to 1999 | **0.92( 0.90- 0.95)*** | 1.00 ( 0.98- 1.01) |
| 2000 to 2004 | **Reference** | **Reference** |
| 2005 to 2009 | **0.79( 0.73- 0.85)*** | **0.83 ( 0.77- 0.89)*** |
| 2010 to 2014 | **0.61( 0.53- 0.71)*** | **0.70 ( 0.62- 0.79)*** |
| 2015 to 2019 | **0.63( 0.55- 0.73)*** | **0.72 ( 0.63- 0.82)*** |

*p<0.001

Table 2. Autointoxication

The differences in period effects between uncorrected and corrected data are highlighted in red.

| **Autointoxication** | | |
| --- | --- | --- |
| **Period** | **North (incorrect)** | **North (correct)** |
| 1980 to 1984 | **0.63( 0.52- 0.77)*** | **0.71 ( 0.62- 0.83)*** |
| 1985 to 1989 | **0.61( 0.51- 0.72)*** | **0.68( 0.60- 0.78)*** |
| 1990 to 1994 | **0.60( 0.49- 0.74)*** | **0.67 ( 0.57- 0.78)*** |
| 1995 to 1999 | **0.73( 0.62- 0.86)*** | **0.77( 0.69- 0.87)*** |
| 2000 to 2004 | **Reference** | **Reference** |
| 2005 to 2009 | **0.73( 0.62- 0.85)*** | **0.77( 0.67- 0.88)*** |
| 2010 to 2014 | **0.58( 0.48- 0.71)*** | **0.66( 0.56- 0.77)*** |
| 2015 to 2019 | **0.57( 0.46- 0.70)*** | **0.67( 0.56- 0.78)*** |
| **Period** | **Northeast (incorrect)** | **Northeast (correct)** |
| 1980 to 1984 | 1.02( 0.91- 1.14) | **1.18( 1.08- 1.28)*** |
| 1985 to 1989 | 0.94( 0.87- 1.01) | 0.95( 0.91-1.004) |
| 1990 to 1994 | **0.86( 0.83- 0.90)*** | **0.79( 0.76- 0.81)*** |
| 1995 to 1999 | **0.83( 0.80- 0.86)*** | **0.76 (0.73- 0.78)*** |
| 2000 to 2004 | **Reference** | **Reference** |
| 2005 to 2009 | **1.39( 1.28- 1.51)*** | **1.50( 1.42-1.58)*** |
| 2010 to 2014 | 1.03( 0.93- 1.13) | **1.08( 1.01- 1.17)*** |
| 2015 to 2019 | **0.76( 0.68- 0.84)*** | **0.78( 0.72- 0.85)*** |
| **Period** | **Southeast (incorrect)** | **Southeast (correct)** |
| 1980 to 1984 | **1.80 ( 1.67- 1.94)*** | **1.60( 1.51- 1.69)*** |
| 1985 to 1989 | **1.09( 1.04- 1.15)*** | **1.06 (1.01-1.28)*** |
| 1990 to 1994 | **0.77( 0.73- 0.82)*** | **0.82( 0.77- 0.87)*** |
| 1995 to 1999 | **0.74( 0.70- 0.77)*** | **0.82(0.80- 0.85)*** |
| 2000 to 2004 | **Reference** | **Reference** |
| 2005 to 2009 | **1.34 ( 1.29- 1.38)*** | **1.24 (1.20 -1.29)*** |
| 2010 to 2014 | **1.32( 1.22- 1.43)*** | **1.33( 1.25- 1.43)*** |
| 2015 to 2019 | **1.19( 1.11- 1.28)*** | **1.10( 1.03- 1.18)*** |
| **Period** | **South (incorrect)** | **South (correct)** |
| 1980 to 1984 | **1.88( 1.71- 2.08)*** | **2.03 ( 1.88- 2.20)*** |
| 1985 to 1989 | **1.29( 1.16- 1.43)*** | **1.39 ( 1.27- 1.53)*** |
| 1990 to 1994 | 0.99( 0.89- 1.11) | **1.08 ( 1.01- 1.15)*** |
| 1995 to 1999 | **0.93( 0.88- 0.98)*** | 0.96 ( 0.92- 1.01) |
| 2000 to 2004 | **Reference** | **Reference** |
| 2005 to 2009 | **1.12( 1.04- 1.20)*** | **1.15 ( 1.09- 1.22)*** |
| 2010 to 2014 | **1.34( 1.18- 1.51)*** | **1.45( 1.36- 1.55)*** |
| 2015 to 2019 | **1.73( 1.55- 1.93)*** | **1.91( 1.74- 2.10)*** |
| **Period** | **Midwest (incorrect)** | **Midwest (correct)** |
| 1980 to 1984 | **2.23( 1.80-2.76)*** | 1.10 ( 0.99- 1.22) |
| 1985 to 1989 | **2.63( 2.13- 3.24)*** | 0.89 ( 0.80- 1.00) |
| 1990 to 1994 | **3.08( 2.46- 3.85)*** | **0.81 ( 0.72- 0.92)*** |
| 1995 to 1999 | **1.42( 1.30- 1.56)*** | **0.89 ( 0.85- 0.95)*** |
| 2000 to 2004 | **Reference** | **Reference** |
| 2005 to 2009 | **1.27( 1.20- 1.34)*** | 0.97 ( 0.89- 1.07) |
| 2010 to 2014 | **2.18( 1.87-2.54)*** | 0.95 ( 0.85- 1.06) |
| 2015 to 2019 | **3.93( 3.02- 5.12)*** | 0.95 ( 0.84- 1.07) |

*p<0.001

**Table 3. Firearm**

The differences in period effects between uncorrected and corrected data are highlighted in red.

| **Firearm** | | |
| --- | --- | --- |
| **Period** | **North (incorrect)** | **North (correct)** |
| 1980 to 1984 | 0.77( 0.54- 1.09) | 0.86( 0.63- 1.17) |
| 1985 to 1989 | 1.05( 0.77- 1.43) | 1.17( 0.89- 1.54) |
| 1990 to 1994 | 1.30( 0.91- 1.86) | **1.35(1.03- 1.78)*** |
| 1995 to 1999 | 1.13( 0.92- 1.39) | 1.20( 0.93- 1.54) |
| 2000 to 2004 | **Reference** | **Reference** |
| 2005 to 2009 | **0.95( 0.74- 1.22)*** | 0.96(0.86- 1.07) |
| 2010 to 2014 | 0.95(0.73- 1.23) | 1.03(0.84- 1.26) |
| 2015 to 2019 | 0.96(0.62- 1.47) | 1.12(0.77- 1.64) |
| **Period** | **Northeast (incorrect)** | **Northeast (correct)** |
| 1980 to 1984 | **0.69(0.54- 0.86)*** | **0.70(0.58- 0.86)*** |
| 1985 to 1989 | **0.80(0.65- 0.97)*** | **0.79(0.67- 0.94)*** |
| 1990 to 1994 | 1.07( 0.87- 1.31) | 1.01( 0.84 -1.19) |
| 1995 to 1999 | **1.46(1.22- 1.75)*** | **1.30(1.11- 1.52)*** |
| 2000 to 2004 | **Reference** | **Reference** |
| 2005 to 2009 | **0.79( 0.73- 0.85)*** | **0.82( 0.77- 0.88)*** |
| 2010 to 2014 | **0.79( 0.68- 0.92)*** | **0.80( 0.70- 0.91)*** |
| 2015 to 2019 | 0.83( 0.63- 1.10) | 0.79 ( 0.62- 1.01) |
| **Period** | **Southeast (incorrect)** | **Southeast (correct)** |
| 1980 to 1984 | **0.86( 0.75- 0.98)*** | **0.75 ( 0.68- 0.83)*** |
| 1985 to 1989 | **1.03( 0.92- 1.16)*** | **0.73( 0.66- 0.80)*** |
| 1990 to 1994 | **1.17( 1.04- 1.32)*** | **0.82( 0.75- 0.90)*** |
| 1995 to 1999 | **1.16 (1.04 -1.30)*** | **1.05( 1.01- 1.11)*** |
| 2000 to 2004 | **Reference** | **Reference** |
| 2005 to 2009 | **0.94( 0.89- 0.99)*** | **0.81(0.76- 0.86)*** |
| 2010 to 2014 | **0.97( 0.88- 1.05)** | **0.74( 0.69- 0.80)*** |
| 2015 to 2019 | **1.01( 0.85- 1.19)** | **0.75( 0.67- 0.84)*** |
| **Period** | **South (incorrect)** | **South (correct)** |
| 1980 to 1984 | **0.55( 0.50- 0.60)*** | **0.68( 0.60- 0.77)*** |
| 1985 to 1989 | **0.66( 0.62- 0.70)*** | **0.71 ( 0.64- 0.79)*** |
| 1990 to 1994 | **0.79( 0.77- 0.82)*** | 0.90 ( 0.80- 1.00)* |
| 1995 to 1999 | **0.94( 0.91- 0.96)*** | **0.75( 0.69- 0.81)*** |
| 2000 to 2004 | **Reference** | **Reference** |
| 2005 to 2009 | **0.88( 0.83- 0.94)*** | **0.81 (0.78-0.84)*** |
| 2010 to 2014 | **0.88 (0.83- 0.94)*** | **0.75 (0.69 -0.81)*** |
| 2015 to 2019 | **0.50( 0.46- 0.54)*** | **0.71( 0.61- 0.83)*** |
| **Period** | **Midwest (incorrect)** | **Midwest (correct)** |
| 1980 to 1984 | **0.53 ( 0.41--0.68)*** | **0.66 ( 0.53- 0.82)*** |
| 1985 to 1989 | **0.76( 0.63 -0.92)*** | **0.82 (0.68- 0.99)*** |
| 1990 to 1994 | **1.11 ( 0.88- 1.39)*** | 1.10 ( 0.91- 1.33) |
| 1995 to 1999 | **1.39 ( 1.16- 1.67)*** | **1.40 ( 1.18- 1.66)*** |
| 2000 to 2004 | **Reference** | **Reference** |
| 2005 to 2009 | **0.78( 0.71- 0.85)*** | **0.80 ( 0.75- 0.86)*** |
| 2010 to 2014 | **0.72( 0.61- 0.84)*** | **0.79 ( 0.69- 0.91)*** |
| 2015 to 2019 | **0.68 ( 0.51- 0.90)*** | 0.80 ( 0.61- 1.05) |

*p<0.001

**Reference**

1. Brasil. Ministério da Saúde. Departamento de Informática do Sistema Único de Saúde. Sistema de Informação sobre Mortalidade [Internet]. Brasília (BR): Ministério da Saúde; 2014. http://sim.saude. gov.br/default.asp. 19.
2. Mello JMH, Gotlieb SLD, Laurenti R. The national mortality information system: problems and proposals for solving them I—Deaths by natural causes. Rev bras epidemiol. 2002; 5:197–211. 1. <https://doi.org/10.1590/S1415-790X2002000200007>
3. Queiroz BL, Freire FHMA, Gonzaga MR, Lima EEC. Completeness of death-count coverage and adult mortality (45q15) for Brazilian states from 1980 to 2010. Rev Bras Epidemiol. 2017; 20(supl 1):21–33. https://doi: 10.1590/1980-5497201700050003
4. Szwarcwald CL, Morais Neto OL, Frias PG, Souza Junior PRB, Escalante JJC, Lima RB, et al. Busca ativa de óbitos e nascimentos no Nordeste e na Amazonia Legal: estimação das coberturas do SIM e do Sinasc nos municípios brasileiros. In: Brasil. Ministério da Saúde. Saúde Brasil 2010: uma análise da situação de saúde e de evidências selecionadas de impacto de ações de vigilância em saúde. Brasília: Ministério da Saúde; 2011. Pp. 79–98.
5. Dantas ESO, Farias YMF, Resende EB, Santos GWS, Silva PG, Meira KC. Estimates of suicide mortality in women residents in northeast brazilian states from 1996 to 2018. Ciencia & saude coletiva.2021; 26:4795-4804. https://doi: 10.1590/1413-812320212610.29552020
6. Soares Filho AM, Cortez-Escalante JJ, França E. Review of deaths correction methods and quality dimensions of the underlying cause for accidents and violence in Brazil. Cien Saude Colet. 2016;21:3803-3818. https://doi: 10.1590/1413-812320152112.13682015
7. Rockett IRH, Hobbs G, Leo D, Stack S, Frost JL, Ducatman AM, Kapusta ND, Walker RL. Suicide and unintentional poisoning mortality trends in the United States, 1987-2006: two unrelated phenomena? BMC Public Health. 2010;10:705. https://doi: 10.1186/1471-2458-10-705.
8. Santos SA, Legay LF, Aguiar FP, Lovisi GM, Abelha L, Oliveira SP. Tentativas e suicídios por intoxicação exógena no Rio de Janeiro, Brasil: análise das informações através do linkage probabilístico. Cad Saude Publica; 2014;30:1057-1066. https:// doi: 10.1590/0102-311x00054213.
9. Brasil, Ministério da Saúde, Secretaria de Vigilância em Saúde. Suicídio: saber, agir e prevenir. Perfil epidemiológico das tentativas e óbitos por suicídio no Brasil e a rede de atenção à saúde [Internet]. 2017 [cited 2022 sep].  <http://portalarquivos2.saude.gov.br/images/pdf/2017/setembro/21/2017-025-Perfil-epidemiologico-das-tentativas-e-obitos-por-suicidio-no-Brasil-e-a-rede-de-atencao-a-saude.pdf>.]
10. Lima EEC, Queiroz BL. Evolution of the deaths registry system in Brazil: associations with changes in the mortality profile, under-registration of death counts, and ill-defined causes of death. Cadernos de saúde pública.2014; 30:1721-1730. https:// doi: 10.1590/0102-311x00131113.

11. Cerqueira D, Ferreira H, Bueno S. (Coord.) Atlas da violência 2020. São Paulo: Ipea; FBSP; IJSN; 2020.

12. Cerqueira D, Ferreira H, Bueno S. (Coord.) Atlas da violência 2021. São Paulo: Ipea; FBSP; IJSN; 2021.

13. Souza ER, Meira KC, Ribeiro AP, Santos J, Guimarães RM, Borges LF, et al. Homicídios de mulheres nas distintas regiões brasileiras nos últimos 35 anos: análise do efeito da idade-período e coorte de nascimento. Cien Saude Colet. 2017;22(9):2949-62.

1) Tabulate and summarize the demographics of the study population, if available in the database, such as the age distribution.

The information system utilized in this study is the Mortality Information System of the Department of Informatics of the Brazilian Unified Health System (SIM/DATASUS). This database does not provide demographic data on women from the Brazilian regions under investigation. However, we sought this information from the Brazilian Institute of Geography and Statistics (IBGE) and believe it is important to include these data in the methodology, so that non-Brazilian readers can assess the sociodemographic differences between these regions.

**Table 2. Sociodemographic indicators and gender inequality indicators in Brazil and its major geographic regions, selected years (2018-2022).**

| **Region** | **Variables** | | |
| --- | --- | --- | --- |
|  | **Population (n)^a^** | **60 or older (%)^b^** | **0 to 9 years old^c^** |
| North | 17.354.884 | 5.36 | 8.17 |
| Northeast | 54.658.515 | 8.05 | 6.75 |
| Southeast | 84.840.113 | 9.98 | 5.83 |
| South | 29.937.706 | 9.77 | 6.04 |
| Midwest | 16.289.538 | 6.87 | 7.08 |
| **Region** | **Population density (inhabitants per km²)^d^** | **Connected to the sewage system (%)^e^** | **Illiterate (%)^f^** |
| North | 4.51 | 24.41 | 8.20 |
| Northeast | 35.21 | 43.06 | 14.20 |
| Southeast | 91.70 | 86.68 | 3.90 |
| South | 51.91 | 67.73 | 3.50 |
| Midwest | 10.14 | 54.27 | 5.10 |
| **Region** | **Literacy rate, female (male) (%)^g^** | **Annual Household Income per Capita USD ($)^h^** | **Female/male earnings ratio^i^** |
| North | 93.0 (94.6) | 2,771.60 | 0.93 |
| Northeast | 87.5 (84.6) | 2,570.50 | 0.87 |
| Southeast | 96.6 (96.9) | 5,084.50 | 0.74 |
| South | 96.4 (97.0) | 4,993.30 | 0.73 |
| Midwest | 95.2 (95.1) | 4,672.80 | 0.76 |

Note: ^a^Total population according to the 2022 Census (https://censo2022.ibge.gov.br/panorama/); ^b^Percentage of the female population aged 60 or over according to the 2022 Census (https://censo2022.ibge.gov.br/panorama/);^c^Percentage of the female population aged 0 to 9 according to the 2022 Census (https://censo2022.ibge.gov.br/panorama/);^d^Population density **(**inhabitants per km^2^) according to the 2022 Census(https://censo2022.ibge.gov.br/panorama/); ^e^Percentage of the Connected to the sewage system according to the 2022 Census(https://censo2022.ibge.gov.br/panorama/);^f^ Percentage of people aged 15 or over who are illiterate according to the 2022 Census(https://censo2022.ibge.gov.br/panorama/);^g^Adult literacy rate (female and male), being the percentage of people aged 15 and above who can both read and write with understanding a short simple statement about their everyday life (BRAZIL, 2021);h Monthly Household Income per Capita (BRAZIL, 2018);^i^ Female-to-male earnings ratio based on average earnings (BRAZIL, 2021)

2) I think the use of a heat map to depict the distribution of different suicide methods among women would be a much clearer and more intuitive way to visualize the results of the study.

We appreciate the reviewer’s suggestion, and we conducted tests with our findings, and the presentation of the data in a heatmap proved to be suitable for summarizing the descriptive data of the specific rates by age group, method, and region (Figure 1). Furthermore, it was also effective in presenting the results of the period effect after adjusting the APC models (Figure 7).

Figure 1. Suicide rates per 100,000 women, by perpetration method, age group, and region. Brazil, 1980-2019.

Figure 7. Risk of suicide in women according to period, adjusted for the age and cohort effect, by method of perpetration, and region. Brazil, 1980-2019.

3) All the figures in the text seem like they could be further optimized to make them more aesthetically pleasing.

We appreciate the reviewer’s suggestion. We have redone all the figures using the R programming language, utilizing the libraries ggplot2, reshape2, gridExtra, scales, patchwork, and ggpubr, which enhanced the definition and layout of the figures. Below is the list of figure titles:

1. Figure 1. Suicide rates per 100,000 women, by Perpetration method, age group, and region. Brazil, 1980-2019.
2. Figure2. Suicide rates per 100,000 women, smoothed by triennial moving averages, according to Perpetration method and region. Brazil, 1980-2019.
3. Figure 3. Suicide rates by hanging/strangulation/suffocation, according to cohort, age, and region. Brazil, 1980-2019.
4. Figure 4. Suicide rates by autointoxication, according to cohort, age, and region. Brazil, 1980-2019.
5. Figure 5. Suicide rates by firearm, according to cohort, age, and region. Brazil, 1980-2019.
6. Figure 6. Suicide rates per 100,000 women adjusted for the period and cohort effect, by age group, method of perpetration, and region. Brazil, 1980-2019.
7. Figure 7. Risk of suicide in women according to period, adjusted for the age and cohort effect, by method of perpetration, and region. Brazil, 1980-2019.
8. Figure 8. Risk of suicide in women according to cohort, adjusted for the age and period effect, by region. Brazil, 1980-2019.
9. Figure 9. Risk of suicide in women according to cohort, adjusted for the age and period effect, by region. Brazil, 1980-2019.

4) The descriptions of the results in each section seem somewhat confusing and lengthy, please summarize the findings in summary terms.

We appreciate the reviewer’s suggestion. We have reorganized and summarized the description of the results, highlighting the key findings. The principal restructuring was carried out in the writing of the results concerning the age, period, and cohort effects of the APC models.

Below are the revised results.

**Reviewer #3:** 
OK.

We have restructured the description and presentation of the results in accordance with Reviewer 2's suggestions. We believe these changes have improved the clarity of this section of the manuscript, as well as the quality of the figures.

**In the text**

**Exploratory Analyses**

From 1980 to 2019, Brazil reported 49,997 suicides by hanging, strangulation/suffocation (55.73%), autointoxication (32.71%), and firearms (11.57%). After adjusting for misclassification and underreporting of death records, there was a 34.62% increase in recorded suicides by the studied methods. The highest percentage increase in suicide rates was observed during the 1980s and 1990s, particularly in the North and Northeast regions (S2 Table).

The highest suicide rates by hanging, strangulation, and suffocation (HSS) and firearm (FA) were observed in the South region, 2.42 and 0.60 per 100,000 women, respectively. Suicides by autointoxication (AUT) exhibited higher mortality rates in the Midwest (1.52 suicides per 100,000 women). In the Southeast region, we observed the lowest rates of suicide by HSS and AUT, respectively, 0.85 and 0.77 per 100,000 women, while the Northeast showed the lowest suicide rates by FA (0.13 suicides per 100,000 women) (Table 2).

**Table 2.** Suicide rates per 100,000 women, by Perpetration method, region, and quinquennium. Brazil, 1980-2019.

**Age effect**

Suicide rates by hanging, strangulation, and suffocation in women, according to age groups, show differences among the Brazilian regions. In the Northeast and Southeast, there was a positive gradient in the magnitude of the rates, with a peak incidence in the 50-54 age group. The South region exhibited a peak incidence among elderly women (70-74 years). In the North and Midwest regions, there was a negative gradient in the magnitude of suicide rates with increasing age, with the highest rates observed among adolescent women (15 to 19 years) (Fig1).

The suicide rates by autointoxication in the North, Northeast, and Midwest regions were higher among adolescents aged 15 to 19 years, with a progressive decline as age increased. In the South and Southeast regions, there was a progressive increase in suicide rates up to the fourth decade of life (Fig1). In all locations, we observed higher rates of firearm suicide among women in their second decade of life, followed by a progressive decline with advancing age (Fig1).

**Figure 1. Suicide rates per 100,000 women, by Perpetration method, age group, and region. Brazil, 1980-2019.**

**Period effect**

Over the forty-year study period, we observed a progressive increase in suicide rates by hanging, strangulation, and suffocation beginning in the mid-2000s in the North, Southeast, and Midwest regions, and from the mid-1990s in the South region (Fig. 2, Table 2). In contrast, suicide rates by firearm exhibited a declining trend across all regions starting in the late 1990s (Fig. 2, Table 2).

Suicides due to autointoxication also exhibited a temporal trend of decreasing mortality rates across all Brazilian geographic regions. In the North and Central-West regions, a reduction in suicide rates was observed beginning in the early 2000s. In the Northeast and Southeast, the decline in rates began from 2009 onwards. In the South, a reduction in suicide rates due to self-intoxication was noted from the 1990s onward, with a subtle increase observed in the final years of the historical series (Fig. 2, Table 2).

**Figure2. Suicide rates per 100,000 women, smoothed by triennial moving averages, according to Perpetration method and region. Brazil, 1980-2019.**

**Cohort effects**

Suicide rates by cohort and age group for suicides involving hanging, strangulation, or suffocation indicate an increase in rates among women born since the 1950s who are between 50 and 54 years old (Fig. 3). Conversely, for suicides due to autointoxication, there was a reduction in rates starting with the 1940-1944 cohort (age group 60 to 64 years) across all regions except the Northeast, where the decrease was observed in the 1965-1969 cohort (age group 40 to 44 years). In the North region, there was an increase in rates among women from the 1940-1944 cohort (ages 60 to 64) (Fig. 4). Regarding suicides by firearm, we observed a progressive reduction in rates among women from the 1930-1934 cohort who are between 50 and 54 years old (Fig. 5).

**Figure 3. Suicide rates by hanging/strangulation/suffocation, according to cohort, age, and region. Brazil, 1980-2019.**

**Figure 4. Suicide rates by autointoxication, according to cohort, age, and region. Brazil, 1980-2019.**

**Figure 5. Suicide rates by firearm, according to cohort, age, and region. Brazil, 1980-2019.**

**Age-Period-Cohort Models**

The analysis of AIC and deviance indicated that the full model (age, period and cohort) provided a better fit to the data in all regions and means of perpetration (S3 Table).

**Age period**

The age-specific rates adjusted for period and cohort effects for suicides by hanging, strangulation, and suffocation exhibited a progressive increase up to the age group of 50-54 years in the South and Southeast regions. In contrast, in other regions, the peak incidence occurred among women aged 80 years or older (Fig 6).Suicides by autointoxication and firearm displayed an opposite pattern, with a progressive decrease in rates with advancing age, except for suicides by autointoxication in the Northeast, which showed an increase with age (Fig 6).

**Figure 6. Suicide rates per 100,000 women adjusted for the period and cohort effect, by age group, method of perpetration, and region. Brazil, 1980-2019.**

**Period effect**

After adjusting the Age-Period-Cohort (APC) models using the estimable functions method, the trend over the 40-year study period, as measured by the age-drift linear trend, indicated an upward trajectory for suicides by hanging, strangulation, and suffocation in all Brazilian regions except the South, where the trend was stationary. Suicides by autointoxication and firearms exhibited a downward trend in all regions, except for autointoxication suicides in the Northeast, which showed an upward trend (Table 3).

**Table 3.** Linear age-drift trend for female suicides, by means of perpetration and region. Brazil, 1980-2019.

The relative risk by period adjusted for the effect of age and cohort (RR), using the period from 2000 to 2004 as a reference, showed an increase in the risk of death (RR>1, p<0.05) in the five-year periods of the 2000s in the North, Southeast, and South regions. In the Midwest region, there was a reduction in the risk of death (RR<1, p<0.05), while in the Northeast, the reduction was not statistically significant at the 5% level (RR<1, p>0.05) (Fig 7, S4 Table).

For suicides by autointoxication in the five-year periods of the 2000s, there was a statistically significant increase in the risk of death (RR>1, p<0.05) in the Southeast, South, and Northeast regions during the periods 2005-2009 and 2010-2014. In the North, there was a reduction in risk during the period of 2005 to 2019, and from 2015 to 2019 in the Northeast. In the Midwest, the decrease was not statistically significant (RR<1,p>0.05) (Fig 7, S4 Table).

Suicides by firearms showed a statistically significant reduction in the risk of death in the period of 2005 to 2019 in the Southeast and South regions, and from 2005 to 2014 in the Northeast and Midwest. The observed increase in the North region was not statistically significant (RR>1, p>0.05) (Fig 7, S4 Table).

**Figure 7. Risk of suicide in women according to period, adjusted for the age and cohort effect, by method of perpetration, and region. Brazil, 1980-2019.**

**Cohort effect**

In the North, Southeast, and Midwest regions, a protective effect on the risk of suicide by hanging, strangulation, and suffocation was observed (RR<1, p<0.05) compared to the 1950-1954 cohort. Conversely, there was a progressive increase in the risk of death among women born from 1955-1959 (Fig 8, S5 Table). In the South region, all cohorts exhibited a higher risk of suicide compared to the 1950-1954 cohort (RR>1, p<0.05) (Fig 8, S5 Table).

Firearm suicides exhibited an inverse trend compared to suicides by hanging, strangulation, and suffocation, as all regions in Brazil showed a reduction in the risk of death from older cohorts (1900-1904 to 1945-1949) compared to younger generations (1955-1959 to 2005-2009) (Fig 9, S4 Table). In suicides by autointoxication, there was also a reduction in the risk of death among younger cohorts (RR<1,p<0.05), with the exception of the Southeast (1955-1979 cohort) and Northeast (1955-2009 cohort), which showed an increased risk among younger women (RR>1,p<0.05)(Fig 9, S5 Table).

**Figure 8. Risk of suicide in women according to cohort, adjusted for the age and period effect, by region. Brazil, 1980-2019.**

**Figure 9. Risk of suicide in women according to cohort, adjusted for the age and period effect, by region. Brazil, 1980-2019.**

**E)Discussion**

**Reviewer1** #1

- “In developing countries, the XX (1965-1979), late Y (1973-1980), and Millennial (1981-1991) generations are experiencing cohort discontinuity.” Usually, in the literature, Generation X refers to people born between 1965 and 1980, while Generation Y – also known as Millennials – refers to those born from the early 1980s to mid-1990s. Therefore, this sentence might be somewhat confusing.

Response: We appreciate your careful review of our manuscript, we have made the correction.

In the text

The higher risk of suicide by hanging, strangulation, and suffocation among women from younger cohorts may correlate with greater awareness of lethality and availability due to increased access to the internet and social media [69]. Additionally, women from Generation X (1965 to 1980) and Generation Y (1981 to 1996) are part of large cohorts and may experience the phenomenon known as cohort discontinuity [99-101].

- “…a pattern that was maintained when estimating the APC models. drift trend…” 491 a 497
  Please correct the typographical error.

Response: We appreciate your careful review of our manuscript, we have made the correction.

“During the study period, Brazil went through several economic crises [72-74,82]…”

- Please consider explaining a bit more about the mentioned economic crises, especially about the specific timing of these events.

Response: We appreciate your careful review of our manuscript, we have made the correction.

- In the text
- Brazil, between 1980 and 2019, experienced several economic crises that deeply shaped the country's social and economic landscape [92-95]. The prolonged crisis of the 1980s, characterized by high inflation and frequent changes in economic plans and currency, was followed by a recession in the 1990s, despite efforts to stabilize the economy with the Real Plan. In the early 2000s, the country experienced a period of growth, but soon faced a new recession due to the global economic crisis of 2008. From 2014 onwards, Brazil entered a phase of economic decline with a reduction in GDP for fifteen quarters, exacerbated by fiscal adjustment measures that reduced spending on health and education and imposed labor and pension reforms [92-95].
- Supporting Information
  S1. Table
  Please correct the typographical errors, such as:
  “Lon-term consequences…”
  “(Stage 6):- ”
  “aP roportion of…”
  “CID-9 and CID-10 Codes”
- Response: We appreciate your careful review of our manuscript, we have made the correction.

**Reviewer 2**

- Line 370-385, page 18-19: The discussion in this article should be addressed to women in the Brazilian region. Is it possible to find more theories to support the results of this study?

Response: We appreciate the careful review of our manuscript. Based on the suggestions of the three reviewers, we have restructured the entire discussion section. In analyzing our findings, we incorporated Durkheim's (2019) analysis of the impact of economic crises on suicide rates, as well as Case and Deaton's (2015) examination of deaths of despair in the context of economic downturns and fiscal austerity. Additionally, we explored the effect of the cohort discontinuity phenomenon on the rise in suicide among women from younger generations. Furthermore, we discussed the influence of gender-based violence on suicidal tendencies among women. These discussions are detailed in lines 573 to 665..

- Overall: 1) Please integrate and summarize some of the passages according to content and logic.

Response: We appreciate the careful review of our manuscript. Based on the suggestions of the three reviewers, we have restructured the entire discussion section. We divided the discussion into the following sections: main findings; regional differences in the magnitude of the rates; age effect; period effect, where we explore the substitution of methods; cohort effect; recommendations for suicide prevention and control actions based on our findings; and limitations and contributions of the study.

- Based on the findings of this study, what actions or measures can be taken by the relevant authorities or the government to improve the situation of female suicide?

Response: We included the suggestion at the end of the discussion in lines 665 to 680.

**Reviewer #3:**

- Discussion should be more focused on your main results. In lines 236-237 you brought your main goal. So, please, be focused on it. Additionally, you should brought these results to the first paragraph of the discussion.
  In addition, the discussion is confused and needs to be reorganized. First discuss all results about age, methods of suicide and, then, cohort.

Response: We appreciate the careful review of our manuscript. Based on the suggestions of the three reviewers, we have restructured the entire discussion section. We divided the discussion into the following sections: main findings; regional differences in the magnitude of the rates; age effect; period effect, where we explore the substitution of methods; cohort effect; recommendations for suicide prevention and control actions based on our findings; and limitations and contributions of the study.

- Are there no studies in Latin America in the same subject? Why to compare results with other countries with greater sociocultural and economic differences?

Response: We appreciate the valuable contribution. In comparing the age and period effects, we included studies from Ecuador, Latin America and the Caribbean, and El Salvador, as well as Brazil (total female suicides) and Rio de Janeiro (firearm suicides). However, we retained studies from the United States, Lithuania, Korea, Japan, Norway, and Australia because the results regarding age, period, and cohort effects were similar to our findings. We emphasize, however, that there are few studies in Latin American countries that have utilized APC models in suicide research. In a search on PUBMED using the keywords 'suicide,' 'age, period, and cohort effect,' 'age, period, and cohort effects, 159 studies were identified, but only three had conducted APC analysis and were carried out in Brazil.

- I didn’t see the point to the paragraph in lines 441-445.
  No need to mention COVID-19 here.

Response: We agree with the reviewer; therefore, we have removed that discussion from the manuscript.
Where is the limitation of this study?

Response: At the end of the discussion, we added a section titled "Limitations and Contributions of the Study.

- Again, I suggest you discuss about public policies fighting gender inequities in Brazil as possible regional barriers to the policies implementations.
  I didn’t see nothing about policies in fighting gender inequities or promoting women health in Brazil in the introduction and in discussion. It is essential to this paper to address this theme

Response: We appreciate the contribution. We have made the suggested modification in lines 640 to 655.

**Reviewer #3:** 
**F)Conclusion**
It needs to be rebuild answering the main goal of your study.

Response: We appreciate the valuable contribution. We have restructured the conclusion to address the study's objectives.
